# Supplementary material for: Generative design of programmable asymmetric β-barrel nanopores
Source: bioRxiv. 2026 Jun 4:2026.06.04.729630. Preprint. [Version 1] doi: 10.64898/2026.06.04.729630 (PMC13252360; doi:10.64898/2026.06.04.729630)
Supplement: Supplementary file 1 [file media-1.pdf]

Supplementary Information

Materials and Methods

Curation of the distillation set

We initialized our distillation-set curation by mining the "Is It A Barrel?" database for putative single-chain transmembrane β-barrel (TMBB) outer-membrane proteins and retained only sequences shorter than 600 amino acids and having complete amino acid sequences (1,938,935 sequences). To minimize redundancy while maintaining adequate sequence and structural diversity for model training, clustering was performed using sequence similarity rather than structural similarity. We clustered the filtered sequences with MMseqs2 using 70% sequence identity and an 80% alignment coverage threshold, yielding 485,379 clusters. For each cluster representative, we then ran BLASTP against the Alphafold Database (AFDB50)[1] to identify entries whose structures are already available. These (~14,000) were excluded so that the remaining representatives could be structure-predicted with Alphafold2 (with MSAs; ~485,000 single-chain predictions) using all five network parameter sets to account for stochastic variation among predictions. Models with mean predicted Local Distance Difference Test (pLDDT) scores below 75 were excluded to remove low-confidence structures. Predicted structures were next subjected to structural quality and β-barrel topology filtering, resulting in 117,447 curated structures. Native structures can have signalling peptides, flexible linkers and soluble domains on top of the barrels. In some cases, barrels can also be stacked on top of each other. Only the transmembrane barrel portion of each structure was considered for downstream filtering, as described below.

For structural filtering and annotation, we processed each predicted PDB using a Rosetta-based pipeline (https://github.com/baker-laboratory/TMB_pipeline). It first assigned a secondary structure using DSSP and identified β-strand segments above a minimum length. Then, it computed backbone-backbone hydrogen bonds for those segments and built an adjacent strand-strand pairing map to ensure the structure formed a closed barrel with a single chain, thus excluding any potentially oligomeric barrels. We only chose barrels that had a minimum of 8 strands and a minimum of 4 backbone-backbone hydrogen bonds per adjacent strand pair. To isolate the barrel from extraneous domains, we then determine barrel-only residues by aligning Cα coordinates of strand residues to the barrel principal axis and trimmed residues that fall outside the median strand span along Z, effectively removing regions outside the barrel cylinder. For confidence control, we restrict subsequent calculations to residues with pLDDT ≥ 75 . Finally, to verify consistency with transmembrane β-barrel topology, β-strand residues were classified into pore-facing (core) and lipid-facing (surface) positions using Rosetta's LayerSelector, and designs were required to contain predominantly hydrophobic lipid-facing strand surfaces (≥60%). The final distillation set is composed of 117,447 monomeric TMBs. Although all proteins in the dataset share the defining β-barrel fold, their sequence space exhibits substantial heterogeneity in strand number, loop topology, and surface polarity distribution.

RFD2 conditioning and finetuning

The curated distillation set was used to fine-tune and condition RFdiffusion2 (RFD2), a deep learning model for de novo backbone generation. When fed into the RFD2 framework, each structure, whether derived from the distillation set or from the PDB, is converted into a series of tensorized inputs that describe both local and global geometric relationships essential for β-barrel folding.

The secondary-structure (SS) tensor is a one-dimensional tensor (N × 4) in which each residue i is represented by a one-hot vector derived from DSSP assignments: helix (H), strand (E), loop (L), or others (N). Because this information is residue-specific rather than pairwise, the SS tensor encodes local backbone conformations and helps the model distinguish structured β-regions from connecting turns or loops.

Feature-specific binary tensors, represented as one-dimensional tensors (N × 1), are included to flag residues involved in key structural features such as β-bulges and glycine twists. Each feature type is encoded in a separate tensor, where a value of 1 indicates that a residue participates in that specific feature and 0 indicates its absence. They provide information at the per-residue level rather than describing pairwise interactions, allowing the model to learn how the placement of each feature independently influences overall β-barrel geometry and curvature.

The adjacency matrix is represented as a two-dimensional tensor (N × N), where N is the number of residues in the protein. Each element (i, j) encodes the spatial relationship between residues i and j, defined by the distance between their Cα atoms. Specifically, a token of 0 indicates that the residues are not within 8 Å of each other, 1 indicates that they are within 8 Å of each other, and 3 denotes that the residues are engaged in β-strand pairing, forming part of the hydrogen-bonded β-sheet network. A token of 2 represents a masked position, meaning that the relationship between those residues (0, 1, or 3) is hidden from the model. Masked tokens give the model freedom to infer the most likely interaction type during training and promote generalization by forcing it to learn underlying geometric patterns rather than memorize specific pairwise contacts. This 2D representation captures both local and long-range dependencies across the protein structure, allowing the model to learn β-strand registry, pairing patterns, and overall curvature, key determinants of β-barrel topology and closure. The final model is referred to as TMB_RFD2. To summarize these additions, Table S1 lists both the existing RFD2 features and the new key features introduced for β-barrel design, and Supplementary Fig. S8 provides a schematic of the training and inference workflow.

We compared inference using fully unmasked adjacency and secondary-structure (SS) tensors with a partial masking strategy applied to both tensors. Specifically, the masking strategy masked most strand-pairing residues in the adjacency matrix while retaining only a small subset of residues near the center of each strand (S11). Similarly, most residues in the secondary-structure (SS) tensor were masked except for a few central residues within each strand (S11). After

benchmarking both approaches, we found that partial masking produced the highest fraction of designs that passed the geometrical filters used to classify structures as barrels (S11).

TMB_RFD2 was trained for 20 epochs (S6). Training initially emphasized TMB structures from the distillation set, with increasing fractions of PDB monomer and PDB interface structures introduced in subsequent rounds. It accepts as inputs the number of strands, average residues per strand, secondary-structure and adjacency specifications, glycine-kink and bulge positions, symmetry, and motifs (Fig. 1b), enabling generation of diverse non-circular β-barrel topologies that were not achievable with previous models. Sampling a broader range of glycine twist positions and combinations resulted in improved SEC profiles, with a higher fraction of designs exhibiting predominantly monomeric peaks compared to the more limited sampling condition (Table 1 vs. Table S3).

MPNN methods

We extended the effective receptive field of the LigandMPNN from 32 to 96 nearest neighbors, and doubled the dimension of all the node and edge embeddings. To store this additional long-range structural information withoutover-squashing, we doubled the dimension of all the node and edge embeddings. We refer to this model as 96NN_MPNN. Starting from the 96NN_MPNN model, we fine-tuned the PDB-pretrained model on the curated TMB distillation set. This model is referred to as TMB_MPNN.

To enable sequence design conditioned on transmembrane hydrophobic thickness, an approximate hydrophobic thickness of each input was first estimated by geometric analysis of the barrel orientation and residue environment. Each barrel was aligned along the Cartesian z-axis and centered at the origin, with the N- and C-terminal ends oriented toward negative z values. Surface-exposed β-strand residues were identified based on solvent accessibility and secondary structure assignments. A tentative hydrophobic thickness was then calculated as the average distance between aromatic-rich residue rings located on opposite ends of the barrel, or between aromatic- and arginine-rich interfacial rings where present. Residues located within this hydrophobic thickness were labeled as buried (class 2), whereas those within the interfacial aromatic or arginine-rich regions were labeled as interface (class 1).

These per-residue thickness labels were encoded as one-dimensional categorical features and incorporated into the TMB_MPNN architecture. Specifically, they were added as residue-level inputs to both encoder and decoder message-passing blocks, serving as conditioning signals in addition to the standard geometric and sequence embeddings. This modification allowed the network to learn correlations between residue type preferences and their depth within the hydrophobic region of the barrel.

During inference, the user can specify a desired hydrophobic thickness value. The model aligns the generated β-barrel along the z-axis and identifies surface-exposed residues from the cis side up to the specified hydrophobic span. The default code takes the central 75% of residues along this span to be assigned the buried label (2), while the remaining 12.5% on either end are

assigned the interface label (1). These percentages can be modified within the codebase (https://github.com/baker-laboratory/TMB_pipeline). These labels are then provided to TMB_MPNN to guide sequence generation consistent with the target hydrophobic thickness. This checkpoint works best for designing barrels with increased hydrophobic thickness.
Additionally, a bias against serine and threonine in pore-facing residues was introduced by further tokenizing surface-facing residues as token 2 and pore-facing residues as token 0 within the buried region. This checkpoint performs best for designing barrels with conventional hydrophobic thickness compatible with lipid bilayers.

Design of de novo TMBs

Backbones were generated using the NC_TMB_RFD2 and TMB_RFD2 model. Required inputs include the number of β-strands and the average number of residues per strand. These inputs are used to automatically construct partially specified secondary structure and adjacency matrix tensors with a wrapper script (https://github.com/baker-laboratory/TMB_pipeline), which are provided to the diffusion model through the job submission script to guide backbone generation toward desired β-barrel architectures. Optional inputs in TMB_RFD2 allow additional geometric control, including specifying strand positions for glycine twists and β-bulges to increase backbone curvature, defining symmetry constraints, and providing structural motifs for motif scaffolding (https://github.com/baker-laboratory/TMB_pipeline). Glycine twist placement for two twists per structure can be sampled following the pattern (i,i+k) for k≥2, and for four twists per barrel using (i,i+1,k,k+1). To target barrel geometries such as triangular shapes, glycine twists can be placed on three approximately equidistant strands around the barrel, while square-like geometries can be obtained by specifying four evenly spaced strands. The wrapper script (https://github.com/baker-laboratory/TMB_pipeline) then automatically places the glycine twists at the specified strand numbers using residues centered around the middle of each strand, corresponding to five residue positions: nres/2-2, nres/2 - 1,nres/2, nres/2+1, nres/2 +2.
β-bulge placement can be achieved by specifying residues at positions $p_i = i \times nres - 1$ for even-numbered strands i, where nres is the number of residues per strand.
When motifs are provided, the model preserves the specified motif geometry while generating the surrounding backbone.
For designing TMBs with copper-binding sites, a three-residue histidine motif, including the backbone coordinates, was extracted from bovine copper-zinc superoxide dismutase (BSOD) and provided as input to TMB_RFD2.
For generation of symmetric repeat alpha-beta TMB pores from scratch, cyclic symmetry at inference was implemented in TMB_RFD2 in the same manner as RFDiffusion[2]. Thereafter, different symmetries were sampled along with necessary inputs for regular TMB_RFD2 diffusion (C4 and C8 for 16 stranded barrel) but with no chainbreaks specified in the contigs resulting in symmetric repeat output structures. These were then designed first using TMB_MPNN, filtered with Alphafold3 and the helical domain further designed with soluble ProteinMPNN followed by another round of structure prediction by Alphafold3

Protein Expression and Purification

All designs were purified from *E. coli* following a protocol adapted from Vorobieva et al. Designs were ordered as synthetic genes (eBlocks, Integrated DNA Technologies) with compatible BsaI overhangs for cloning into the target vector LM0627 via Golden Gate assembly, with a stop codon introduced prior to the His-tag in LM0627. Golden Gate subcloning reactions were performed in 96-well PCR plates in 1 μL volumes. Reaction mixtures were transformed into chemically competent *E. coli* BL21(DE3) cells, followed by recovery in 100 μL SOC medium, and then distributed into four 96-deep-well plates containing 900 μL auto-induction medium composed of 1% (w/v) tryptone, 1% (w/v) sodium chloride, 0.5% (w/v) yeast extract, 1% (w/v) kanamycin. After overnight incubation at 37°C (20-24 h), cultures were scaled up into 50 mL of autoclaved TB-II medium supplemented with 1% (w/v) kanamycin, 2 mM $MgSO_4$, and 1× 5052 for further expression.

Cells were harvested and lysed. Inclusion body pellets were washed several times with buffers containing 1% Triton X-100 and 1% Brij-35, alternating between the two detergents to remove residual lipids and other contaminants. Each washing step involved resuspension of the insoluble pellet in the appropriate buffer, brief sonication, and incubation for one hour at room temperature or overnight at 4°C.

After washing, pellets were solubilized in GuCl, and 500uL of the denatured protein solution were refolded in 30mL of a buffer containing 25 mM Tris-Cl (pH 8.0), 100 mM NaCl, and 0.1% DPC (dodecyl-phosphatidylcholine). Refolding was carried by spontaneous dilution. These conditions enable hydrophobic β-barrel domains to refold into detergent micelles that mimic the membrane environment.

The refolded samples were incubated overnight at 4°C with gentle shaking, concentrated using 10 kDa molecular-weight cutoff filters, and purified by size-exclusion chromatography (SEC) on a Superdex 200 Increase 10/300 GL (Cytiva) column. Fractions eluting at the expected volume for the monomeric state (~99mL) of the designed pore were pooled, concentrated, and stored at 4°C for subsequent analysis.

Electrophysiological Characterization

Membrane insertion and conductance measurements were performed using the Nanion Orbit 16 TC instrument on MECA chips, which enable up to 16 parallel bilayer recordings. Lipid stock solutions were freshly prepared in dodecane at a concentration of 1 mg/mL, using DPhPC (di-phytanoyl-phosphatidylcholine) lipids for all experiments.

Purified proteins were diluted into a buffer containing 0.05% DPC (~1× CMC), 25 mM Tris-Cl (pH 8.0), and 100 mM NaCl, to a final protein concentration of approximately 5 μg/mL. A small aliquot (~0.5 μL) of this protein solution was added to the cis chamber of the chip containing 200 μL of KCl buffer while lipid bilayers were formed using the Orbit's built-in rotating stir-bar setup. Measurements were conducted at 25°C under symmetric buffer conditions, with either 1 M KCl or 1.5 M KCl present on both sides of the membrane.

Spontaneous membrane insertion was detected as discrete current jumps characteristic of single-channel openings. Raw signals were recorded at a sampling frequency of 10 kHz and downsampled to 100 Hz using an 8-pole Bessel filter. Current traces were processed using a custom Python script (https://github.com/baker-laboratory/TMB_pipeline).
Between runs, MECA chips were washed with multiple rounds of ethanol and water and fully dried before reuse. Conductance profiles, stability over time, and insertion frequency serve as key indicators of membrane compatibility and robustness. Clean, stable current jumps suggest proper β-barrel formation and integration into the lipid bilayer.

For comparison of conductance across different cationic and anionic electrolyte conditions, single-channel electrophysiology measurements were performed in symmetric 1 M electrolyte solutions containing $CaCl_2$, KCl, NaCl, LiCl, KF, KCl, and KI, and conductance values were determined from the resulting current traces.

For testing motif-scaffolded designs containing a copper-binding site within the pore (N10_AP_des5), 2 μL aliquots of 100 mM $CuSO_4$ were added to the cis chamber three times, with incubation periods between additions to allow diffusion to the pore. Control experiments were performed under identical conditions using $CoCl_2$ and K_2SO_4. The same $CuSO_4$ addition protocol was also applied to the mutant construct his_des5_AP_mut1_model.

For testing designs in polymer-lipid hybrid membranes, we used membranes composed of 10% PEO-b-PBO block copolymer and 90% 16-carbon DPhPC lipid.

Circular Dichroism
For screening designs in detergent micelles, the protein/detergent was directly analyzed by CD spectrometry in SEC buffer (25 mM Tris-Cl (pH 8.0), 150 mM NaCl, and 0.1% DPC (dodecyl-phosphatidylcholine)). CD spectra were obtained using a Jasco model J-1500 spectropolarimeter over a wavelength range of 260-190 nm at room temperature. Average CD spectra from three repeats were obtained using a Chirascan Plus (Applied Photophysics).

ssDNA experiments
A 22 nucleotide Thymine repeat ssDNA was purchased from IDT and used for the ssDNA translocation experiments on N14_ML_des8. 2uL of the DNA was added on top of a single pore in a DPhPC bilayer from a 1uM stock solution in water. The bilayer was painted across a symmetric solution of 25mM Tris and 200mM NaCl in a MECA 100uM chip. The applied voltage was changed in increments of 25mV from 50 to 150 mV and the corresponding current recorded at a sampling frequency of 20kHz. The recorded signal was then filtered using a digital 8 pole low-pass bessel filter at 5kHz and simple thresholding based event detection was carried out using custom scripts in python (https://github.com/baker-laboratory/TMB_pipeline). The dwell time histogram was fitted with an exponential distribution and their corresponding means plotted at different applied voltages.

DNA threading experiment

The Oxford Nanopore sequencing kit (SQK-RAD114) was used following their suggested protocol for lambda phage DNA to prepare the Sequencing Buffer (SB) plus DNA mix. A custom in-house PET29b vector backbone was used instead of the lambda DNA. In a MECA 100uM 16 cavity chip, N14_AP_des2 pore was reconstituted in DPhPC lipid bilayers across a symmetric salt solution containing 1M KCl and 25mM Tris at pH 8.0. The solution temperature was increased to 38C using the Orbit16 TC temperature controller and 40uL of the sequencing mix was added near the cavity containing the pore. The added buffer was pipetted up and down a couple of times and the applied voltage was increased to 150mV slowly in steps of 10mV increments. The current signal was recorded at 10kHz sampling frequency and subsequently filtered using a 8 pole low-pass bessel digital filter to 2kHz. The recorded current was not normalised to compare raw current perturbations to the standard ONT R10 pore signal with the same sequencing mix. For both cases, events were first estimated using the PELT algorithm with the ruptures python library. Then a histogram of all the found levels was fitted with a bayesian gaussian mixture model (GMM) with a maximum of 15 states with the sklearn library. Then using a 6 percent threshold for state weights, an optimal number of states was selected for subsequently fitting a hidden markov model (HMM) model. The hmmlearn library in python was used to fit the hmm model to the filtered signal with initialised means of PELT levels from a deterministic GMM model using the optimal number of states as determined above. The filtered signal was divided into 90:10 train:test set for the HMM training and validation. Predicted current states on the validation signal were plotted with shaded regions for each state indicating the square root of their covariance matrices.

JIBE experiment

The monoglyceride JIBE was formulated by dissolving monolinolein (Nu-Chek Prep Inc., MN) with squalane oil (≥ 90%, Sigma Aldrich) to a concentration of 10 mg/mL. Monolinolein has a chain length of 18 carbons with two points of unsaturation at carbon 9 and 12, abbreviated as C18:2 Δ9,12 cis. This mixture was combined with squalene oil (≥ 98%, Sigma Aldrich) and hexadecane oil (99%, Sigma Aldrich) at a volume ratio of 1:1:2, respectively, to a final volume of 0.75 mL. The aqueous phase consisted of Milli-Q water (resistivity ≥ 18.2 MΩ-cm, TOC < 50 ppb, Milli-Q IQ7000, Merck) containing 200 mM KCl (Fisher Chemical) and 30 mM Bis-Tris (ULTROL® Grade, EMD Millipore Corp), adjusted to pH7 with HCl. The aqueous solution was added dropwise to the oil phase at a 1:1 volume ratio to yield a total volume of 1.5 mL. Protein solutions (1-3 µL) were added directly to the oil-water mixture to achieve a lipid-to-protein ratio (LPR) of 1500 (TMB12_3 WT) or 3000 (N10_AP_des2). For the negative controls, 9.38 µL of elution buffer (TBS-DPC) was added in place of protein. The oil-water mixture was vortexed at 3000 rpm for 1 min and centrifuged at 8000 × g for 1 min. Excess oil was removed after centrifugation, yielding a jammed water-in-oil emulsion in which monoglyceride-stabilized droplets formed bilayers at droplet interfaces. A custom three-part device was fabricated using a

Bambu Lab P1P 3D printer with 0.2 mm PLA filament to support the JIBE during voltage-clamp experiments. The assembled device consisted of two outer plates and a central spacer (2 mm thickness) that defined the JIBE chamber. Each outer plate contained a circular aperture with an outer diameter of 10 mm and an inner diameter of 7.5 mm. The apertures were filled with 2% (w/v) agarose hydrogel (Molecular Biology Grade, Fisher Chemical) to a thickness of 2.5 mm. The JIBE was loaded into the central chamber between the hydrogel layers, resulting in a membrane area of approximately 0.78 cm^2. The inner diameter of the hydrogel layers is reduced compared to the outer diameter to prevent the agarose layers from being displaced out of the plates. Fluid reservoirs were secured on both sides of the hydrogel-JIBE-hydrogel assembly. Each reservoir was filled with 2.5 mL of the same aqueous solution used for JIBE preparation (200 mM KCl, 30 mM Bis-Tris, pH7).cElectrical measurements were performed using a patch clamp amplifier (HEKA EPC 10 USB) in a two-electrode configuration. Ag/AgCl electrodes (0.25 mm diameter) were placed in each reservoir on the opposite side of the JIBE membrane, with one connected to the amplifier headstage and the other grounded. To promote protein insertion into the droplet interface bilayers, a constant DC conditioning voltage was applied prior to measurement. Specifically, +1 V was applied for 15 min, followed immediately by -1V for 15 min. This conditioning step resulted in a gradual increase in current over the 30 min period, consistent with increased protein incorporation into the membrane. Omitting this step resulted in significantly lower currents during subsequent measurements. Following conditioning, current-voltage (I-V) measurements were performed by applying a DC voltage sweep from -200 mV to +200 mV in 40 mV increments. Each voltage step was held for 30 s, and current was recorded at a sampling rate of 50 kHz (20 μs) with a 1 kHz Bessel filter applied during acquisition. Membrane conductance was determined from the slope of the linear fit to the I-V relationship.

Filtration experiments with JIBEs

To prepare the JIBEs with lipids, the oil phase was prepared by adding 125 μL squalene, 125 μL 30 mg/mL C18 glycerol monolinoleate lipids in squalene, and 250 μL of hexadecane to a 1.5 mL centrifuge tube. The aqueous phase was prepared as a 1 mM Tris-Cl (pH 8.0) and 10 mM KCl solution. Next, 240 μL of the aqueous phase and 10 μL of 1 mg/mL protein of interest were simultaneously added to the oil phase. The emulsion was immediately vortexed at 3200 rpm (Scientific Industries Vortex-Genie 2) for 5 min, then centrifuged at 5000 rcf for 5 min (Eppendorf™ Centrifuge 5430 R) to form the JIBE.

To prepare the JIBEs with diblock copolymers, the oil phase was prepared by adding 75 μL of 220 mg/mL PB770:PEO183 diblock copolymer in squalene to 175 μL squalene and 250 μL hexadecane in a 1.5 mL centrifuge tube. The aqueous phase was prepared as a 1 mM Tris-Cl (pH 8.0) and 10 mM KCl solution. Next, 240 μL of the aqueous phase and 10 μL of 1 mg/mL protein of interest were simultaneously added to the oil phase. The emulsion was immediately vortexed at 3200 rpm (Scientific Industries Vortex-Genie 2) for 4 minutes and centrifuged at 20,000 rcf (Eppendorf™ Centrifuge 5430 R) for 3 minutes.

To assemble the device for the filtration experiment, 0.5% agarose solutions were made, one with 10 mM KCl and 1 mM Tris-Cl (pH 8.0), and one with 10 mM KCl, 10 mM LiCl, 10 mM NaCl, 10 mM CaCl and 1 mM Tris-Cl (pH 8.0). Immediately before assembly, the temperature of the agarose solutions was 45-55°C. Using a syringe with a removable needle, we filled one plastic chamber well (Figure S24a) with the corresponding agarose solution, then inserted the syringe into the channel and flipped the chamber, releasing agarose into the O-ring and tilting to spread agarose thinly and evenly (~1 mm thickness). After letting agarose dry for 20-30 seconds, the syringe needle was partially untwisted, the chamber flipped over, and remaining agarose pressed out to fill the top of the syringe needle. At this point, the syringe needle was attached to one chamber. We repeated with the second solution and plastic chamber. Using Ag/AgCl electrodes (0.015" diameter) and an ammeter with pico to nanoampere resolution, we checked if the chamber was electrically connected throughout by inserting one electrode into the agarose in the syringe needle and gently touching the other electrode to the agarose surface in the O-ring. If the current did not exceed the upper current threshold (e.g. 200 nA) we remade the chamber setup. We pipetted the clear oil phase off the top of the JIBEs (Figure S24b), cut the top of the JIBEs centrifuge tube, and used flat tip tweezers to wipe JIBEs onto the surface of the agarose pads and O-rings on both plastic chambers (Figure S24c). We assembled the device by placing one plastic chamber into the larger metal chamber with the JIBE layer face up, then placing the other plastic chamber face down so that the O-rings touched. The small metal chamber piece was gently screwed onto the larger metal chamber piece. The positive electrode was inserted into the needle head of the chamber containing the multi-ion solution and a voltage applied to measure conductance (Figure S24d). Applying a constant voltage of 450 mV facilitated protein insertion over time.

Crystallography

All crystallization experiments were conducted using the sitting drop vapor diffusion method. Crystallization trials were set up in 200 nL drops using the 96-well plate format at 20 ˚C. Crystallization plates were set up using a Mosquito LCP from SPT Labtech, then imaged using UVEX microscopes and UVEX PS-256 from JAN Scientific. Diffraction quality crystals formed in 0.05 M Glycine, pH 9, and 55% (v/v) PEG 400 for N10_AP_des7 and in 0.2 M Ammonium chloride, 40% (v/v) MPD for N10_RS_des4.

Diffraction data was collected at the Advanced Photon Source beamline on 24-ID-E and National Synchrotron Light Source II on FMX. X-ray intensities and data reduction were evaluated and integrated using XDS[3] and merged/scaled using Pointless/Aimless in the CCP4 program suite[4]. Structure determination and refinement starting phases were obtained by molecular replacement using Phaser for N10_AP_des7[5] and DIMPLE for N10_RS_des4[6] using the designed model for the structures. Following molecular replacement, N10_AP_des7 was improved using phenix.autobuild[7]; efforts were made to reduce model bias by setting rebuild-in-place to false, and using simulated annealing and prime-and-switch phasing. N10_AP_des7 was refined in Phenix[7]. Model building was performed using COOT[8]. The final model was evaluated using MolProbity[9]. For N10_RS_des4, iterative model building and corrections were performed

manually using COOT[8] following molecular replacement and subsequent structure refinement was performed with CCP4 Refmac5[10]. Initial refinement was conducted using BUSTER[11] to rapidly fix Ramachandran, rotamer, and density fit outliers, refining to convergence. PDB-REDO[12] was used to assess the model quality in between refinements and to fix any rotamer and density fit outliers. Final refinement was carried out in Phenix[7], with manual model building performed in COOT[8]. The model quality was evaluated using MolProbity[9]. Data collection and refinement statistics are recorded in Table S4. Data deposition, atomic coordinates, and structure factors reported in this paper have been deposited in the Protein Data Bank (PDB), http://www.rcsb.org/ with accession code XXXX.

Table S1. Input feature representations used for training TMB_RFD2 model

Features	Dimensions	Description
**1D Features**		
is_β-bulge	2	Binary indicator assigned to a single residue, encoding the presence or absence of a β-bulge at that position.
is_glycine_kink	2	Binary indicator encoding a glycine-mediated twist motif defined over a five-residue local backbone window.
secondary_structures	5	One-hot encoding with five states: helix (0), strand (1), loop (2), mask (3), and small molecule (4).
motif_sequence	79	Amino acid identity representation of the scaffolded motif sequence.
**2D Features**		
adjacency_matrix	4	Pairwise residue–residue encoding with four states: far (0), close (1), mask (2), and β-strand pair (3).
**3D Features**		
motif_structure	3	Three-dimensional Cartesian coordinates of motif residues, extracted exclusively from pore-facing regions during training.

Table S2. Sequences of all experimentally tested designs that exhibited conductance

Table S3. Percent good SEC and percent functional designs from a design campaign using TMB_RFD2 with glycine twists specified on two strands positioned approximately half a barrel apart. For each barrel type, the number of designs with good SEC profiles is reported relative to the total number of designs, and functional success rates are calculated relative to designs with good SEC profiles.

**Designs from TMB_RFD2 (less glycine twist positions sampled)**				
**Barrel type**	**Good SEC**	**Good SEC (%)**	**Functional**	**Functional (%)**
N10	5/12	41.7%	2/12	16.7%
N12	2/16	12.5%	1/16	6.3%
N14	2/10	20.0%	1/10	10.0%

Table S4. Crystallographic data collection and refinement statistics.

	N10_AP_des7 **(PDB ID: XXXX)**	N10_RS_des4 **(PDB ID: XXXX)**
**Data collection**		
Space group	$C\ 2\ 2\ 2_1$	$P\ 4_1\ 2\ 2$
Cell dimensions		
a, *b*, *c* (Å)	87.64, 87.64, 142.50	70.19, 70.19, 220.67
α, β, γ (°)	90, 90, 90	90, 90, 90
Resolution (Å)	71.25 - 4.00 (4.47 - 4.00)	34.66 - 4.00 (4.47 - 4.00)
R_{merge}	2.03 (3.48)	0.38 (0.46)
$I / \sigma I$	5.7 (2.7)	10.1 (8.2)
Completeness (%)	99.8 (99.8)	99.9 (100)
Redundancy	11.9 (11.5)	24.4 (23.8)
$CC_{1/2}$	0.821 (0.556)	0.972 (0.953)
**Refinement**		
Resolution (Å)	46.76 - 4.0 (4.40 - 4.00)	9.99 - 4.0 (4.13 - 4.00)
No. reflections	4843 (1202)	4758 (477)
R_{work} / R_{free}	0.2887 (0.3219) /0.2510 (0.2508)	0.3573 (0.3529) /0.3241 (0.3633)
No. atoms		
Protein	2424	3464
Ligand/ion	n/a	n/a
Water	n/a	n/a
B-factors		
Protein	57	56
Ligand/ion	n/a	n/a
Water	n/a	n/a

R.m.s. deviations		
Bond lengths (Å)	0.002	0.003
Bond angles (°)	0.723	0.72

*Single Crystal used for each data collection. *Values in parentheses are for highest-resolution shell.

A

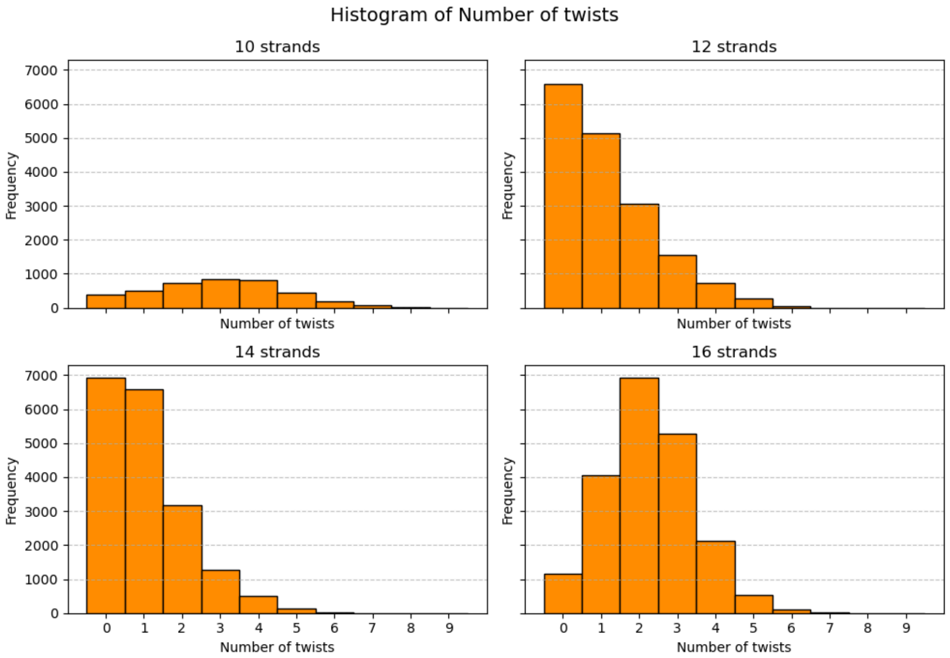
Histogram of Number of twists
10 strands
12 strands
14 strands
16 strands
Frequency
Number of twists
0 1000 2000 3000 4000 5000 6000 7000
0 1 2 3 4 5 6 7 8 9

B

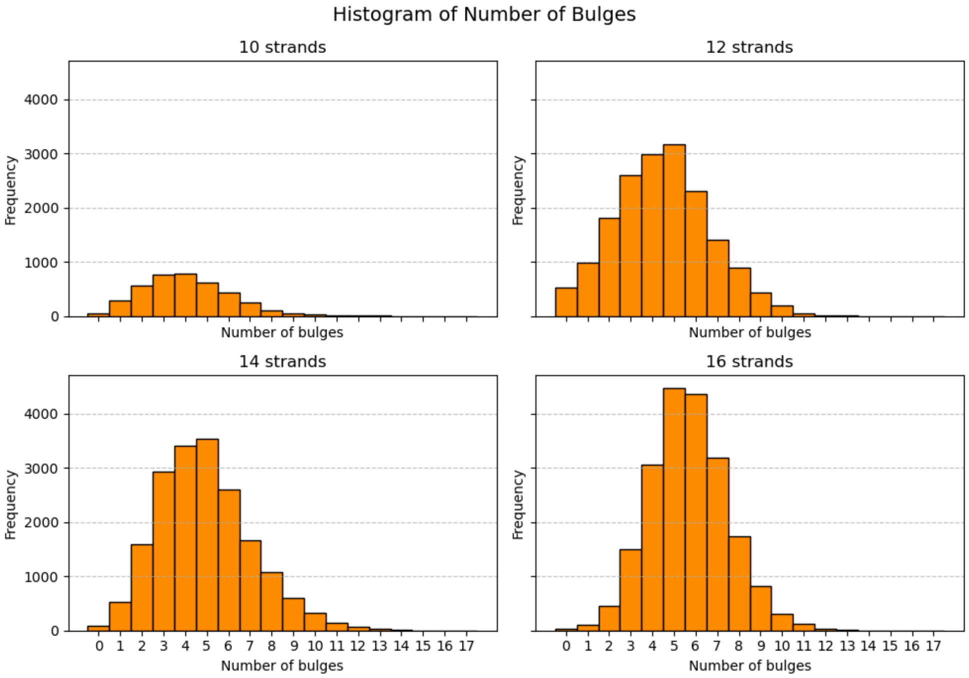
Histogram of Number of Bulges
10 strands
12 strands
14 strands
16 strands
Frequency
Number of bulges
0 1000 2000 3000 4000
0 1 2 3 4 5 6 7 8 9 10 11 12 13 14 15 16 17

Figure S1. (A) Distribution of the number of glycine twists in 10-, 12-, 14-, and 16-stranded TMBs from the distillation set. **(B)** Distribution of the number of β-bulges in 10-, 12-, 14-, and 16-stranded TMBs from the distillation set.

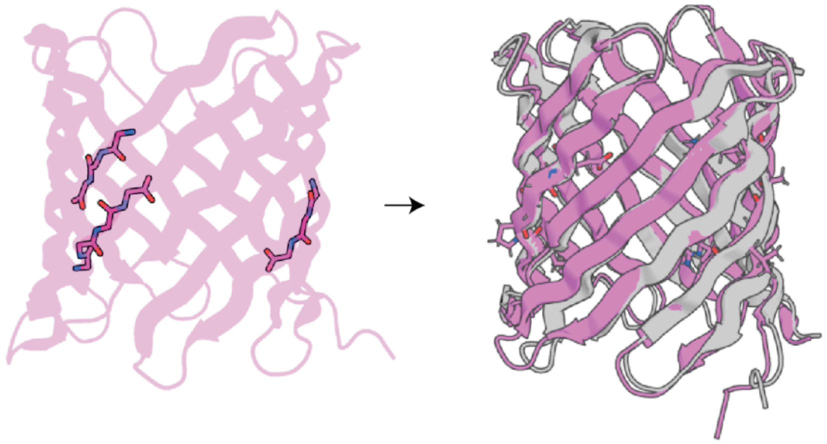

Figure S2. Crystal structure of design made by scaffolding a glycine twist. Motif provided on left in pink, design on right in pink, crystal on right in gray.

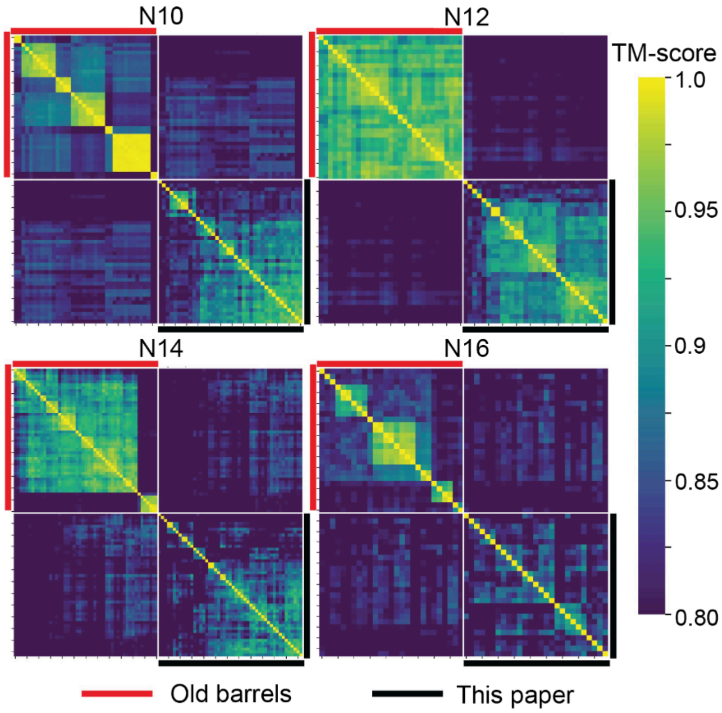

Figure S3. All-by-all pairwise TM-score heatmap for 50 designs from parametric-guided approach and 50 from TMB_RFD2 across four strand numbers. High–TM-score clusters manifest as large yellow diagonal blocks and indicate lower structural diversity within the set.

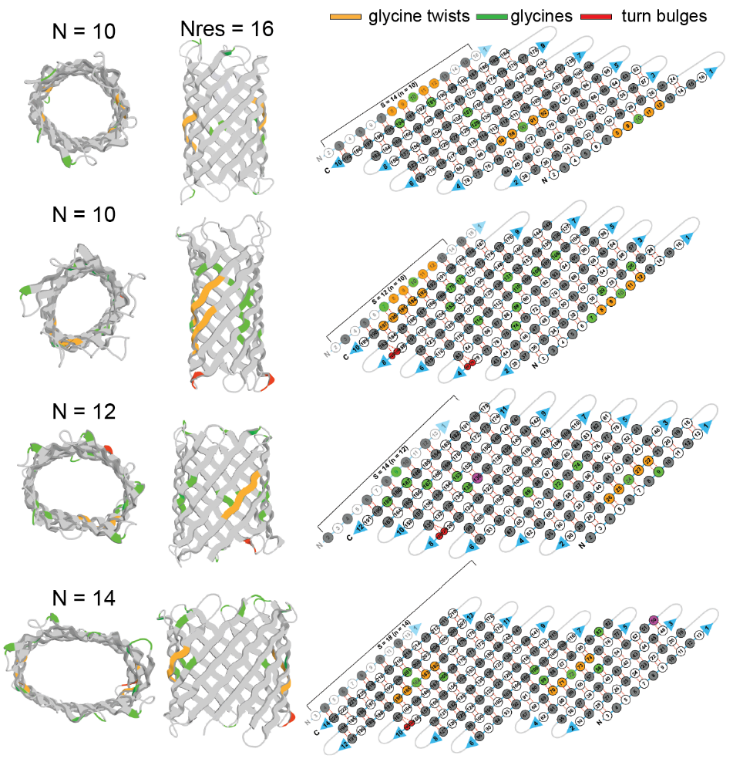

Figure S4. Example outputs of four β-barrels spanning a wide range of strand shear values using only two inputs: strand number (n) and residues per strand (nres). Necklace plots show diverse β-sheet registries and barrel geometries.

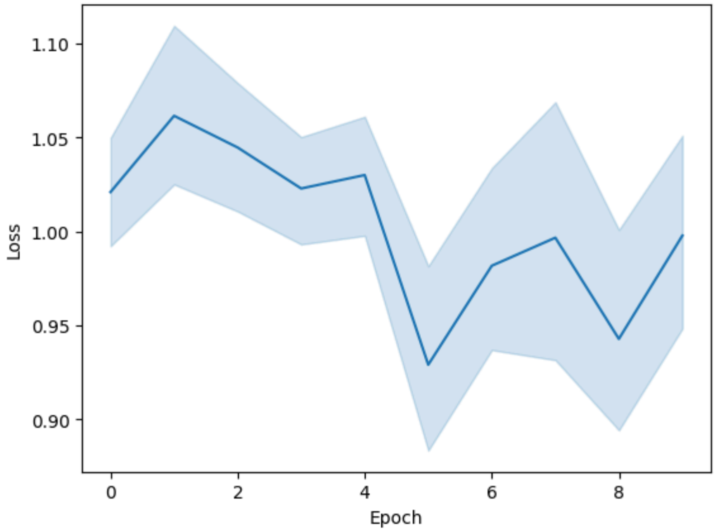

Figure S5. Average training loss per epoch during fine-tuning of TMB_RFD2 on the distillation set with conditioning. Each epoch comprised 24,000 structures with a batch size of 8.

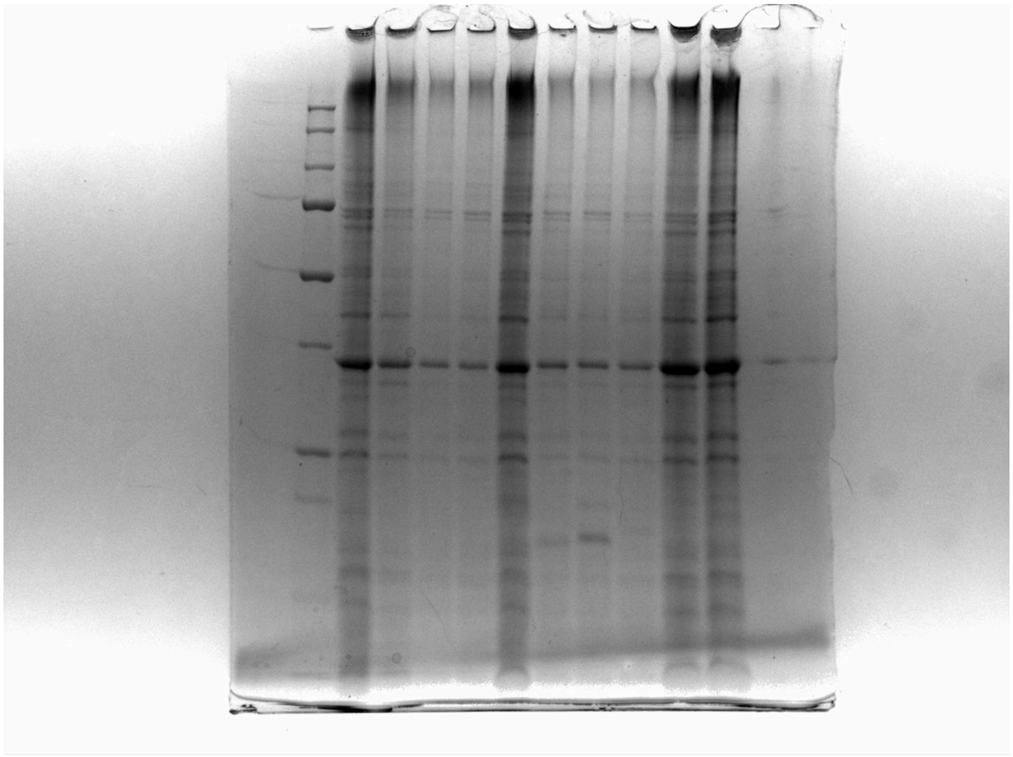

Figure S6. Experimental characterization revealed no/poor expression in *E. coli* for de novo generated TMB sequences using ProteinMPNN

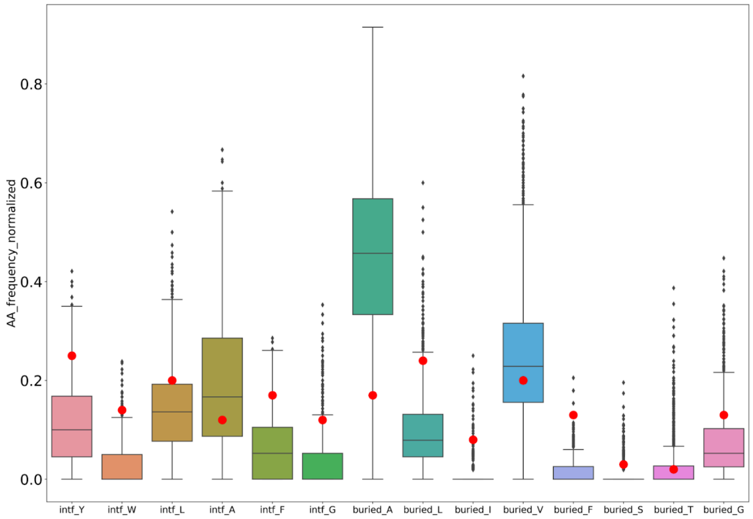

Figure S7. High bias for hydrophobics on the surface of TMB designs using standard ProteinMPNN.

A

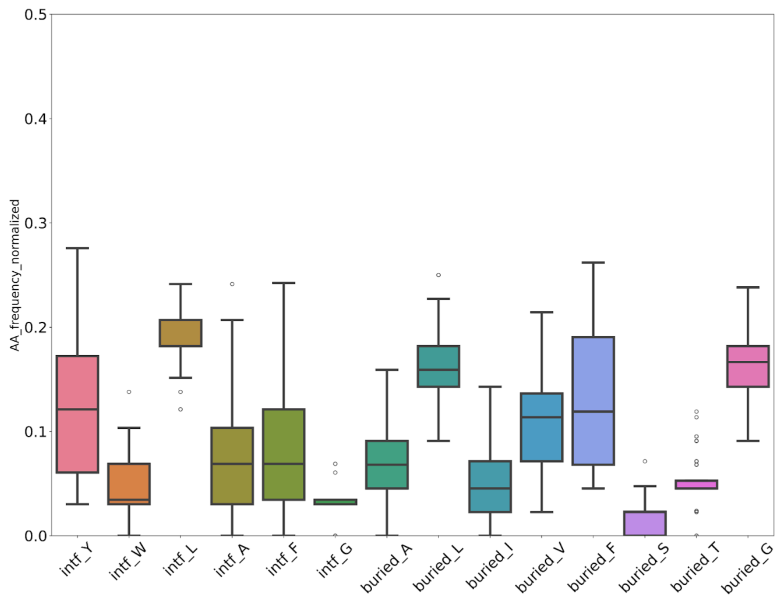

0.5
0.4
0.3
0.2
0.1
0.0
AA_frequency_normalized
intf_Y
intf_W
intf_L
intf_A
intf_F
intf_G
buried_A
buried_L
buried_I
buried_V
buried_F
buried_S
buried_T
buried_G

B

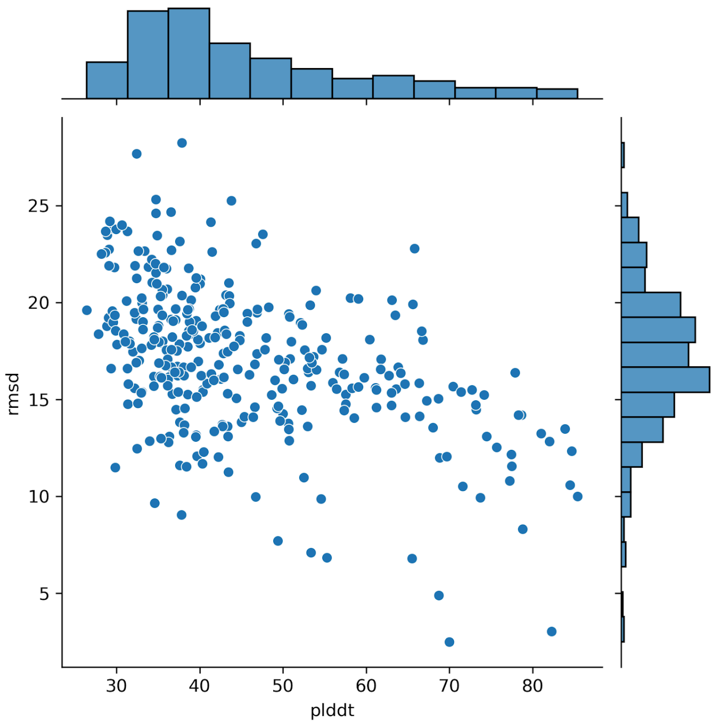

rmsd
25
20
15
10
5
30
40
50
60
70
80
plddt

Figure S8. (A) More uniform distribution of hydrophobics on the surface of TMB structures with finetuned ProteinMPNN on the TMB distillation set. **(B)** Very few predicted barrels using Alphafold for the above mentioned sequences.

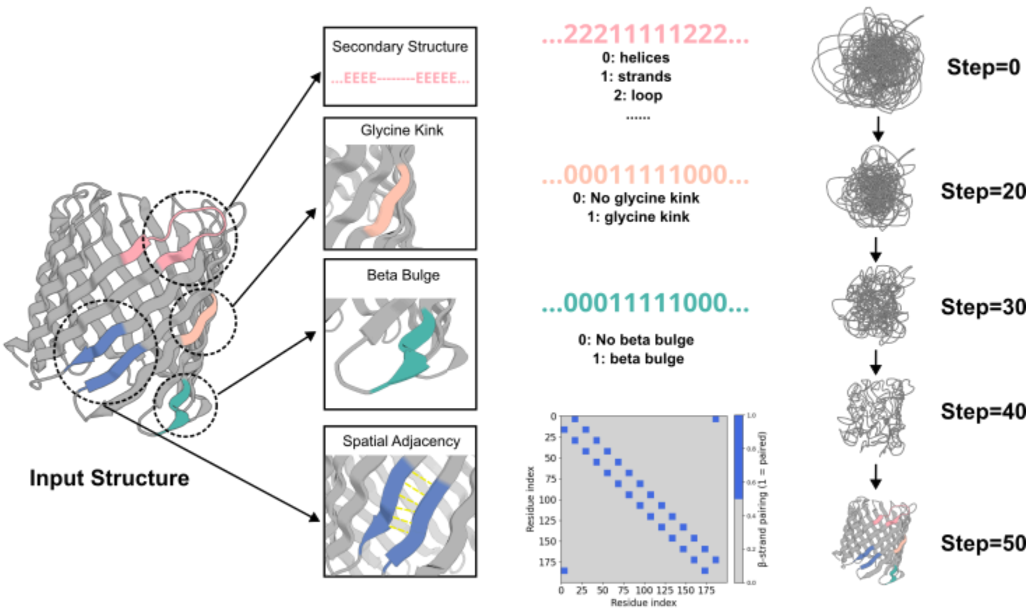

Figure S9. Schematic representation of TMB_RFD2 training features and the inference process. The model is conditioned on multiple structural constraints, including residue-wise secondary structure assignments (helices, strands, and loops), glycine-induced backbone kinks, and β bulges. These local geometric features are complemented by a spatial adjacency matrix (lower center) that defines the global β-strand pairing topology. During inference (right), the model begins from a disordered state at Step 0 and progressively refines the protein backbone through 50 iterative denoising steps, ultimately converging on a folded transmembrane β-barrel structure that satisfies the input architectural requirements.

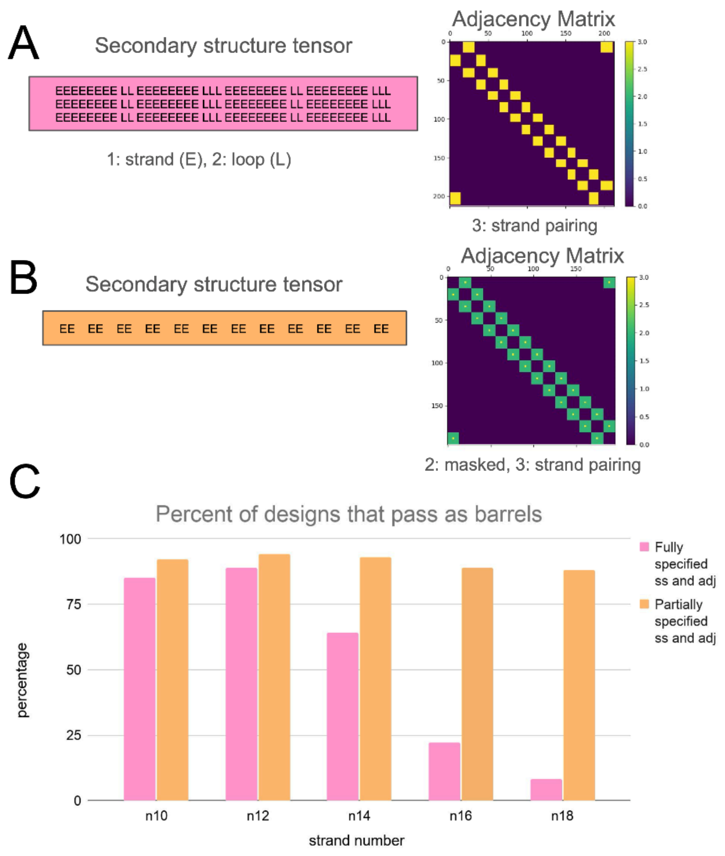

Figure S10. (A) Example input for fully specified secondary structure tensor and adjacency matrix for a 10 stranded barrel (B) Example input for partially specified secondary structure tensor and adjacency matrix for a 10 stranded barrel (C) Comparison of designs that pass as β-barrels when using fully specified versus partially specified secondary structure tensors and adjacency matrices shows that partial specification increases barrel recovery across all strand numbers.

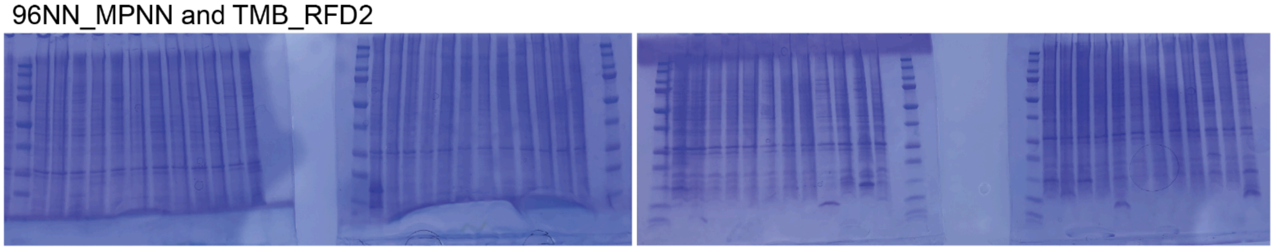

Figure S11. No expression visible on SDS-PAGE gels for designs ordered using 96NN_MPNN on TMB_RFD2 backbones.

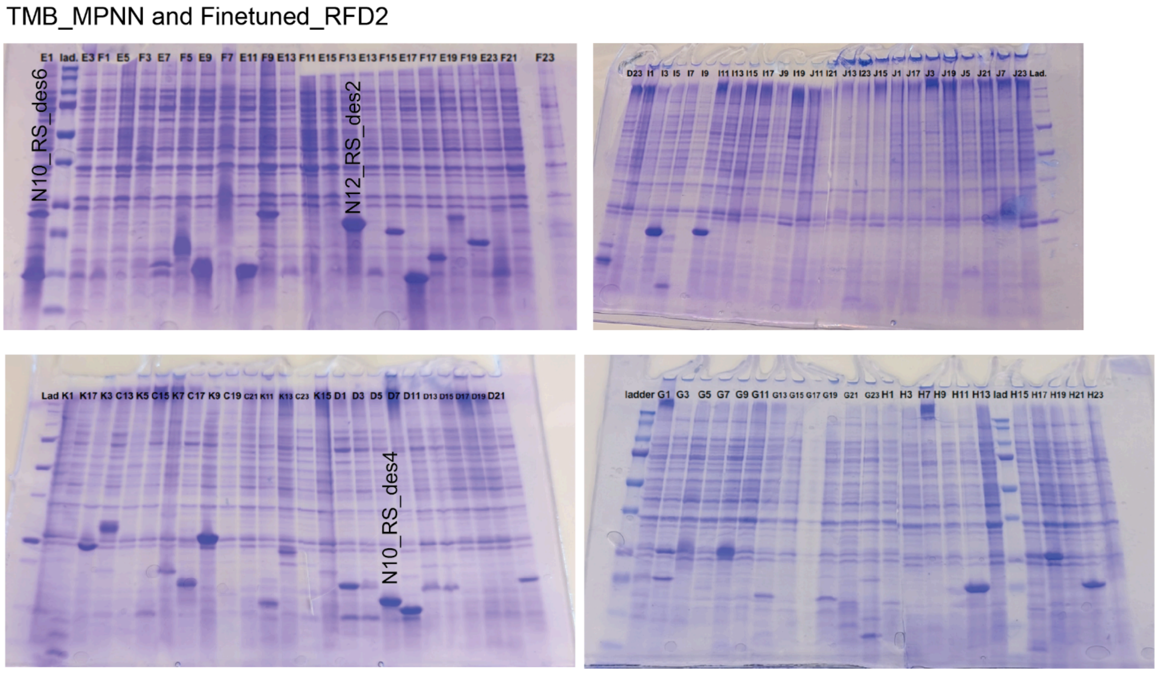

Figure S12. SDS-PAGE gels of designs generated with Finetuned_RFD2, with sequences designed using TMB_MPNN. Functional designs are labeled according to Supplementary Table 2.

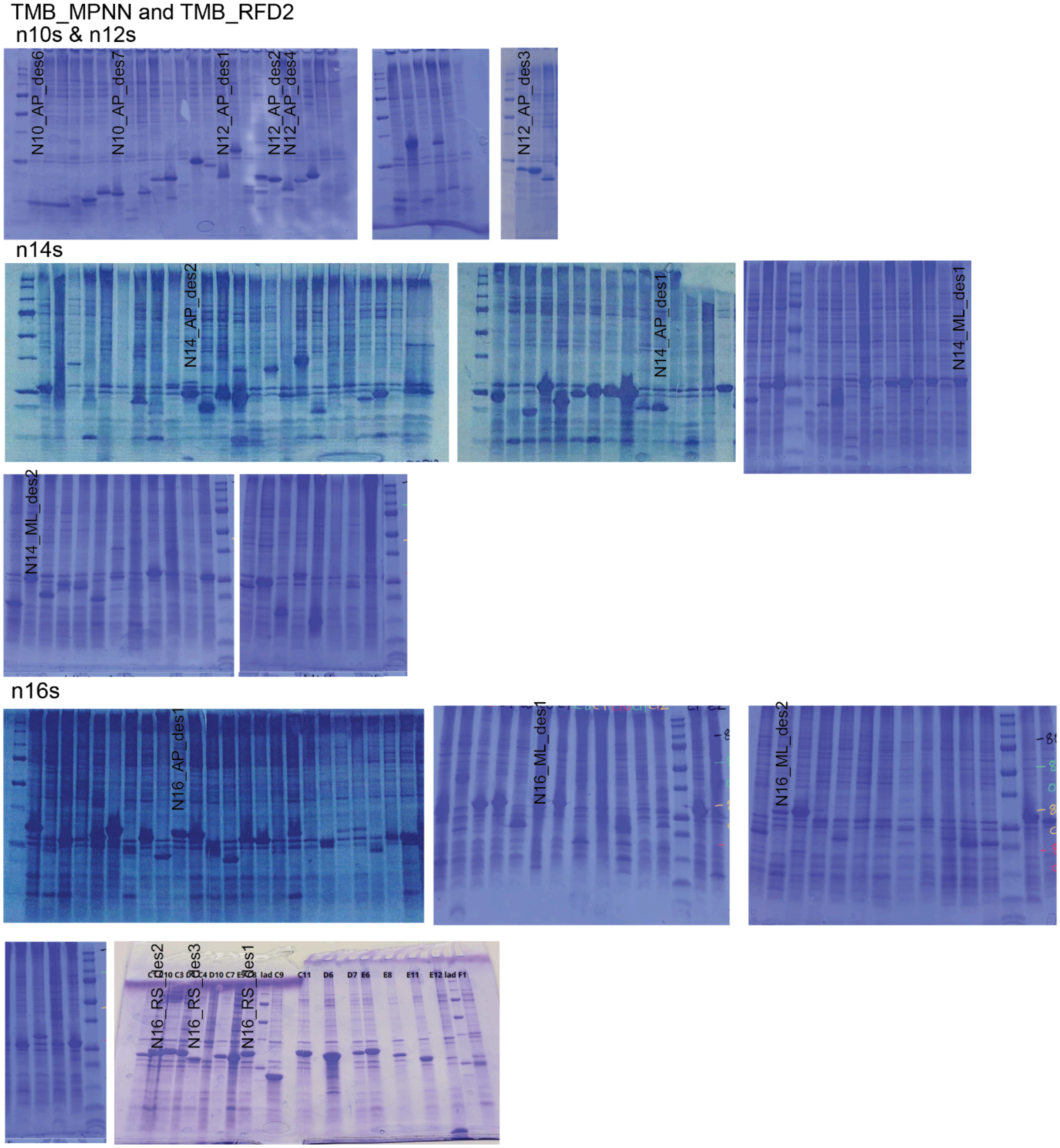

Figure S13. SDS-PAGE gels of designs generated with the RFD2 model trained on the distillation set and conditioned on TMB features and secondary structure, with sequences designed using TMB_MPNN. Functional designs are labeled according to Supplementary Table 2.

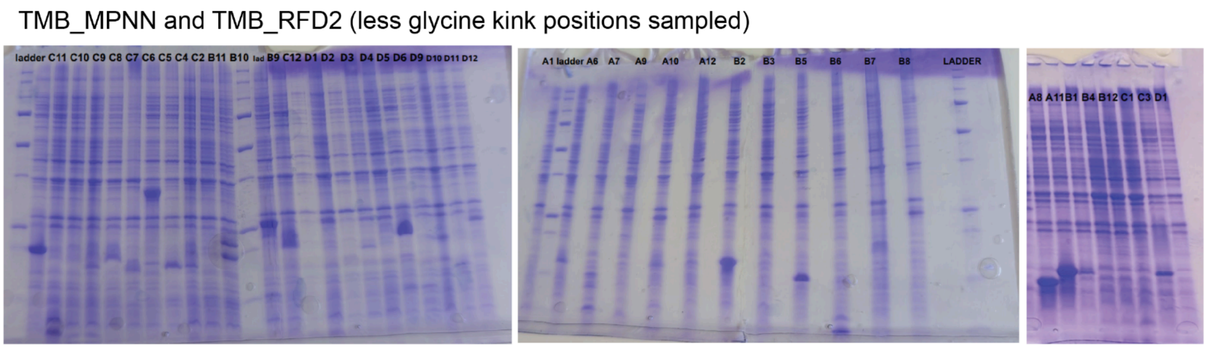

Figure S14. SDS-PAGE gels of designs generated with TMB_RFD2 backbones in which fewer glycine kink positions were sampled during backbone generation, with sequences designed using TMB_MPNN.

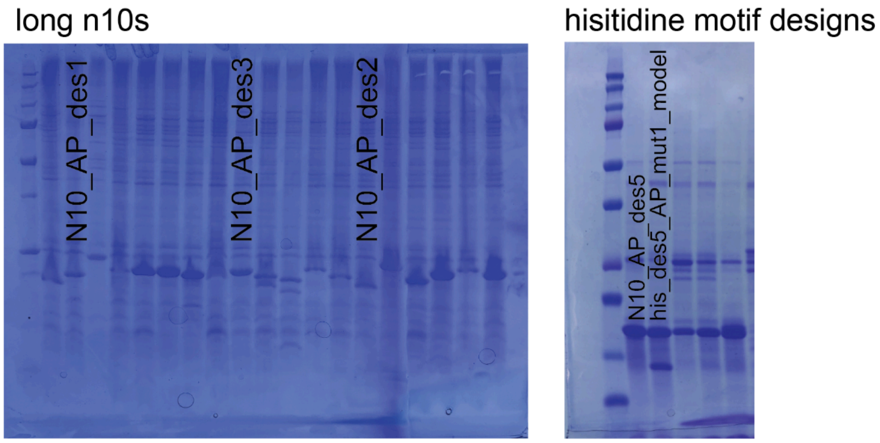

Figure S15. SDS-PAGE gels of designs ordered with conditioned hydrophobic thicknesses from TMB_MPNN on longer TMB_RFD2 backbones, alongside a histidine-motif-scaffolded design and its histidine-motif mutant. Functional designs labeled according to S Table 2.

Finetuned_RFD2 and TMB_MPNN

n10s

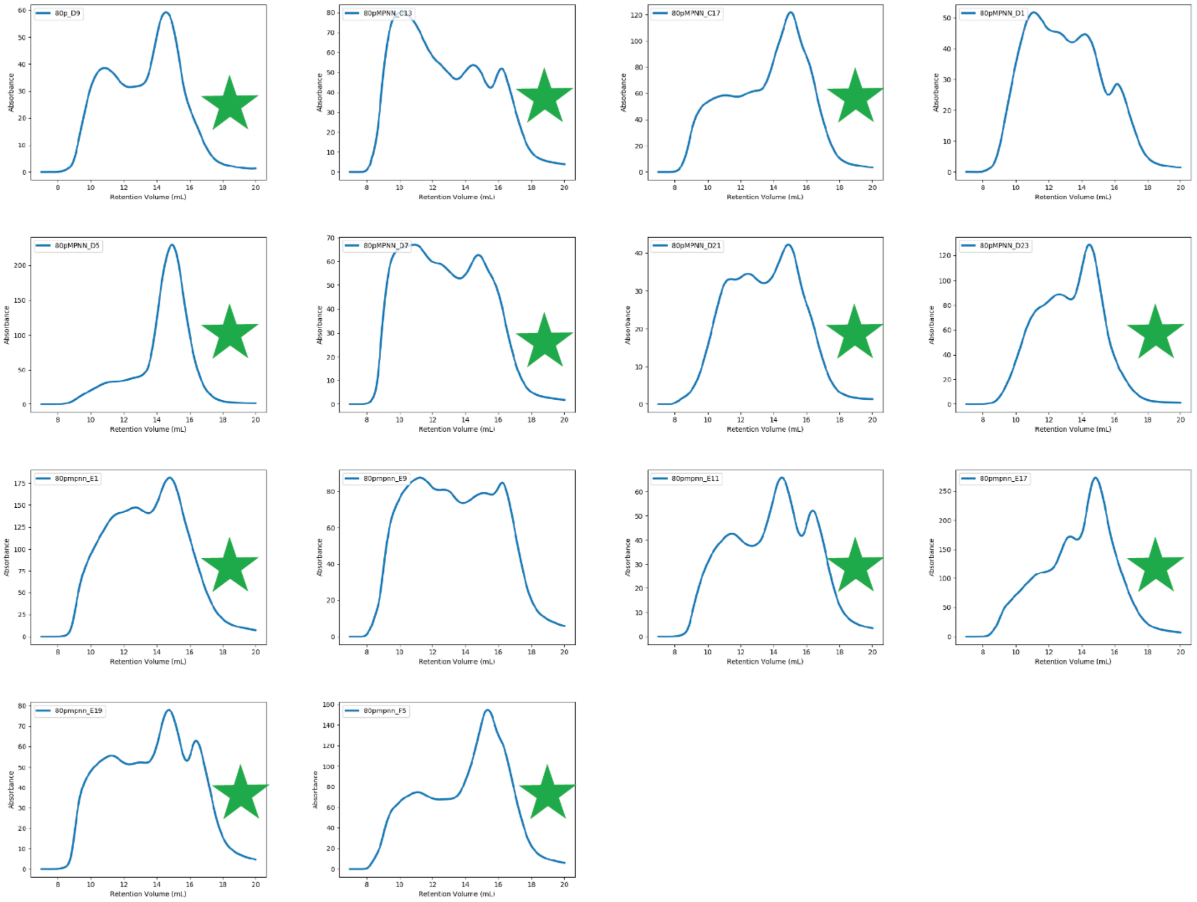

n12s

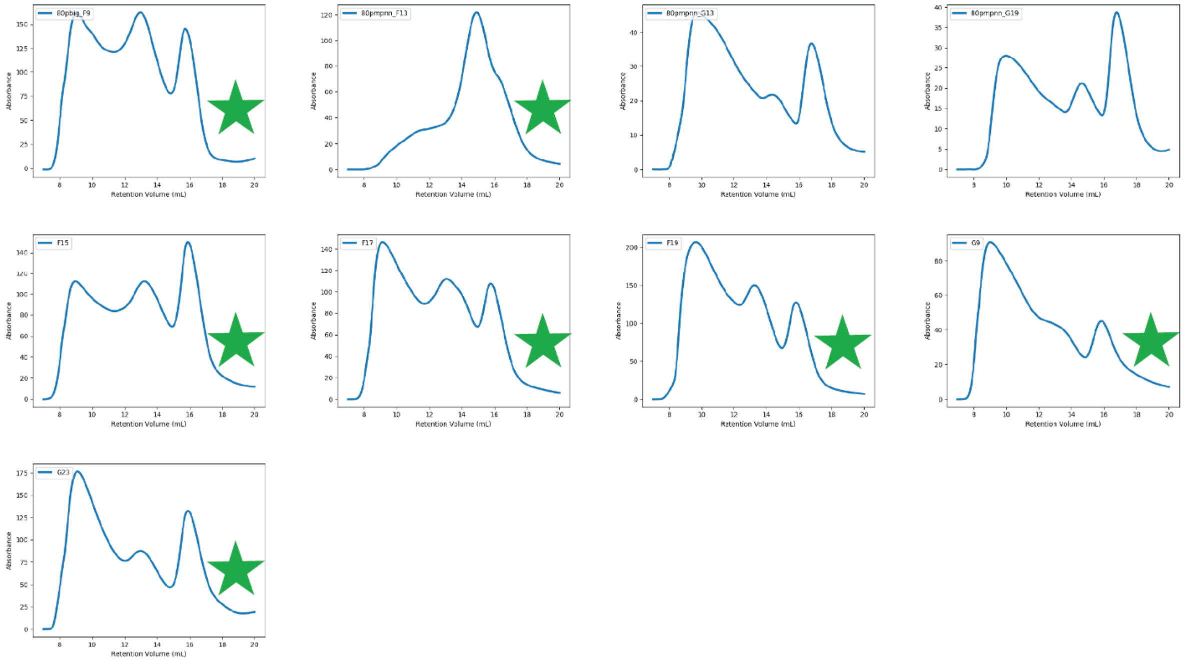

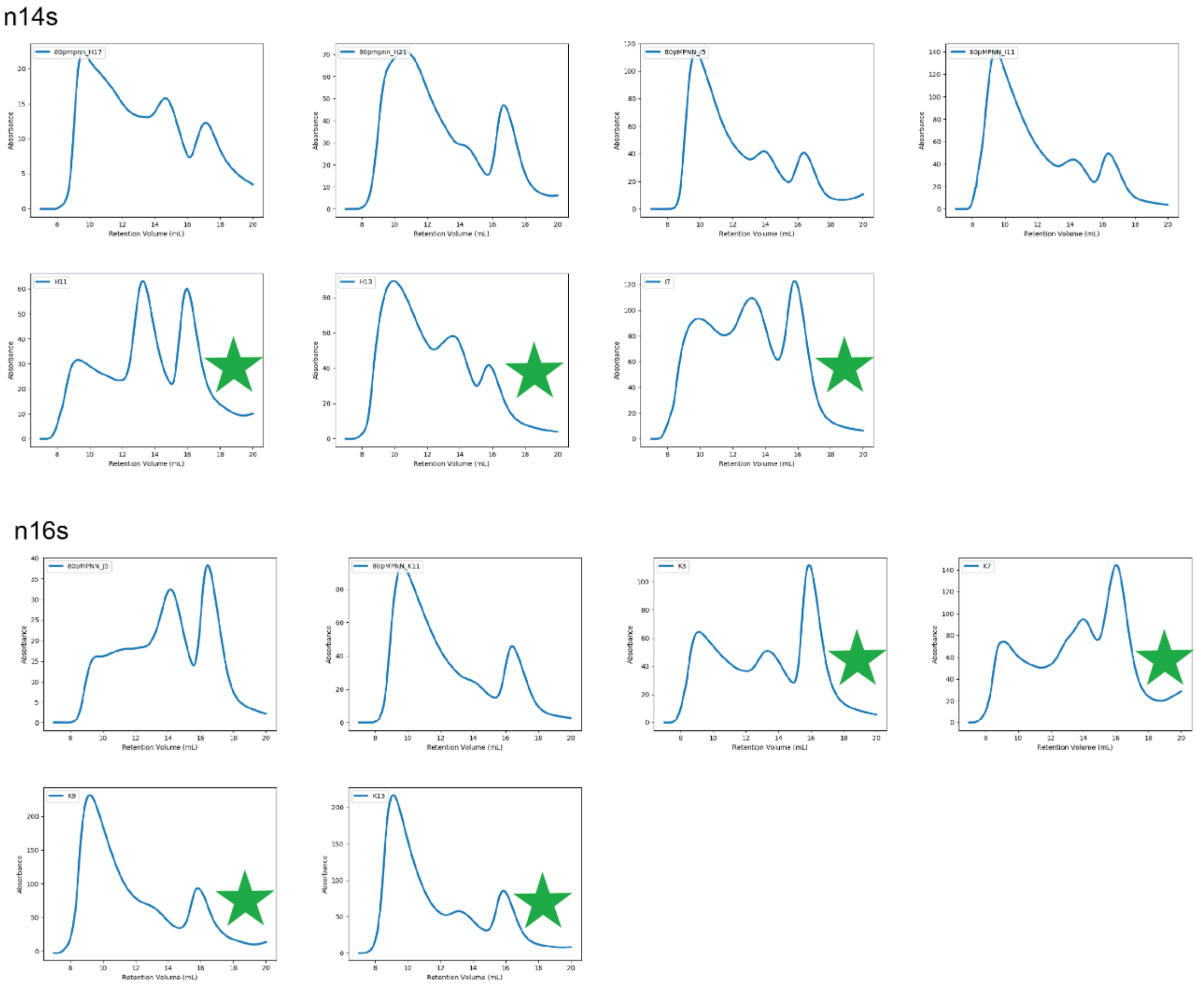

Figure S16. SEC traces of Finetuned_RFD2 and TMB_MPNN designs. Constructs with "good" traces (marked by green stars) were advanced to membrane insertion assays and used to generate Table 1.

TMB_RFD2 and TMB_MPNN

n10s

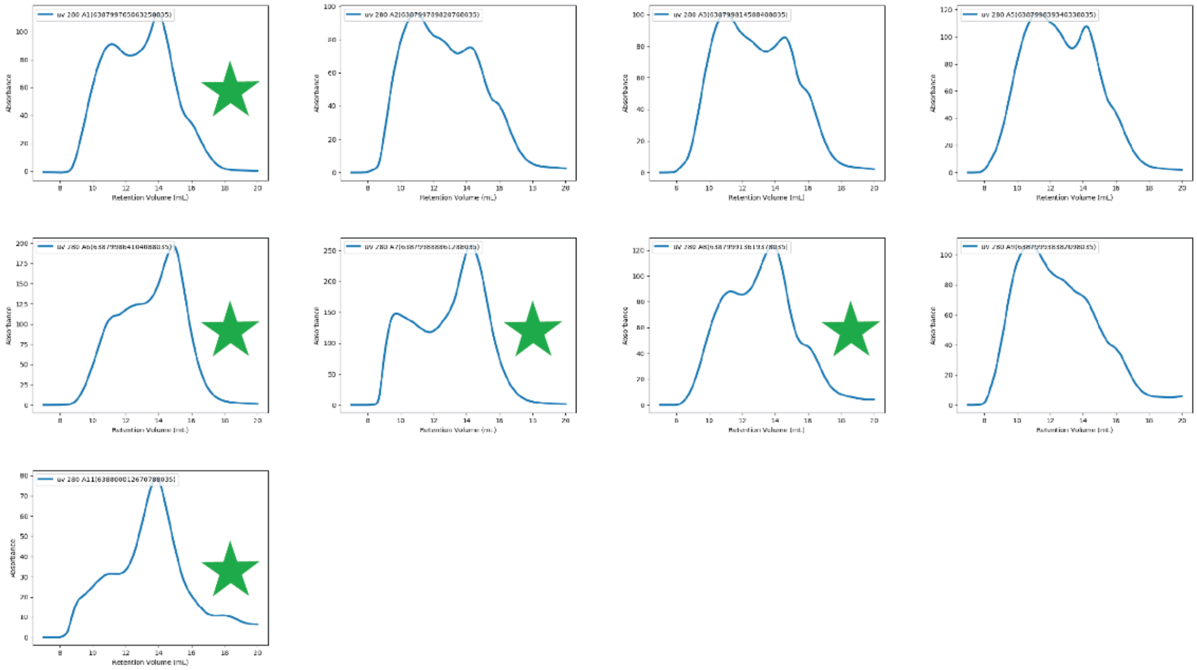

n12s

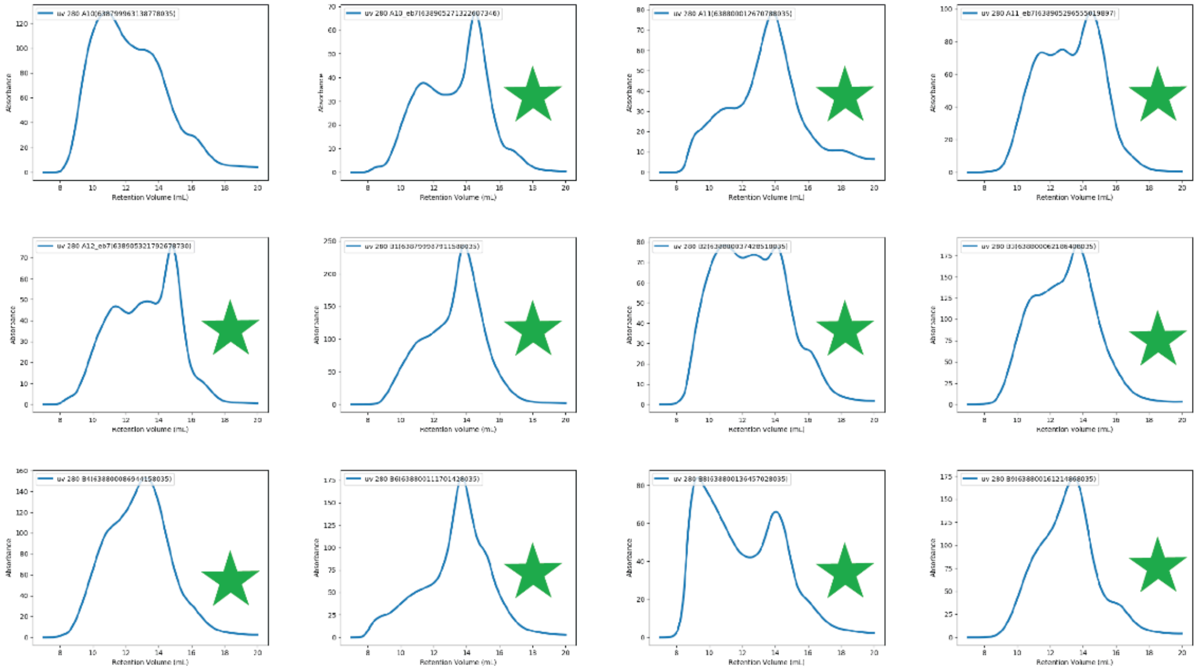

n14s

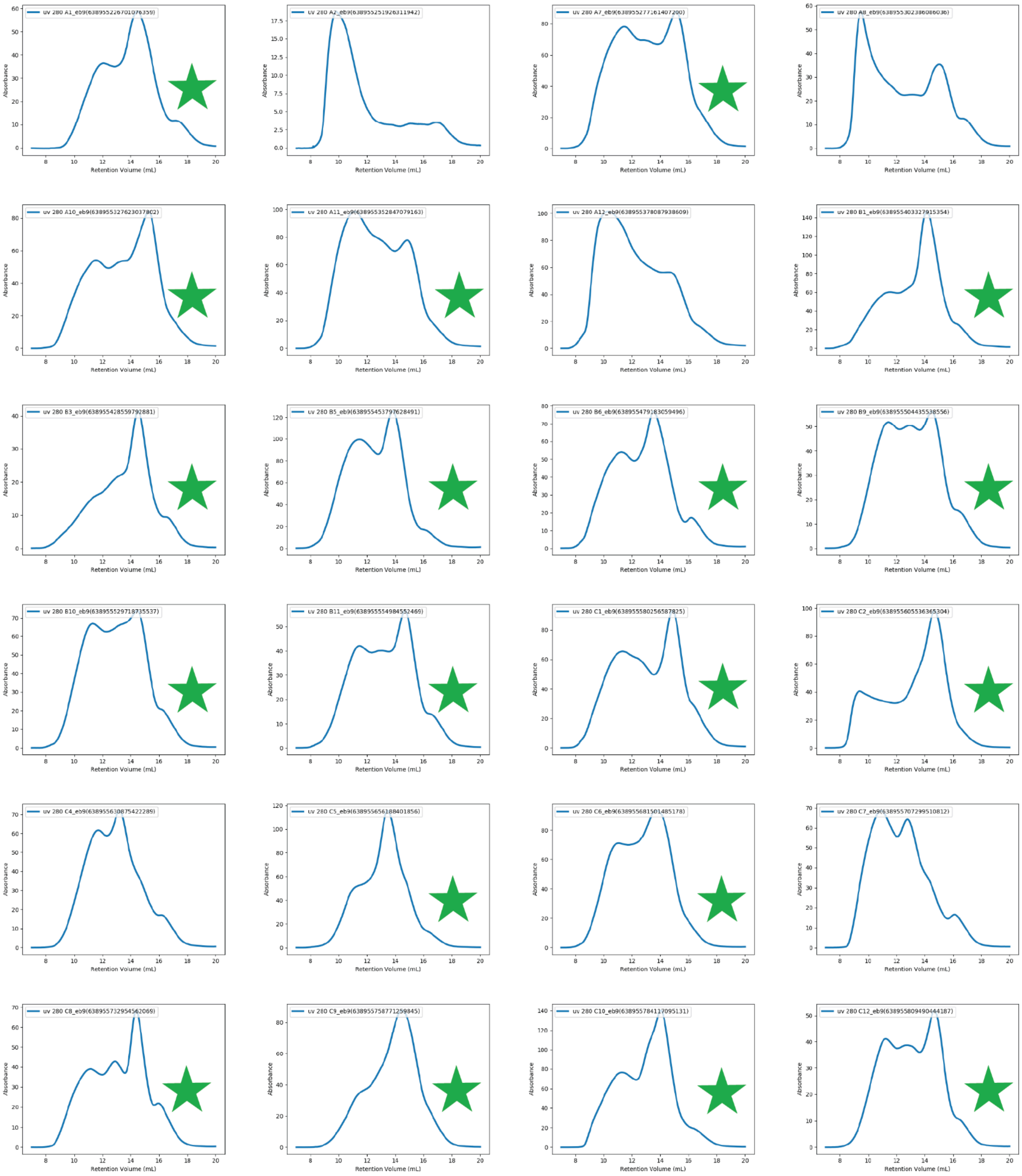

n14s

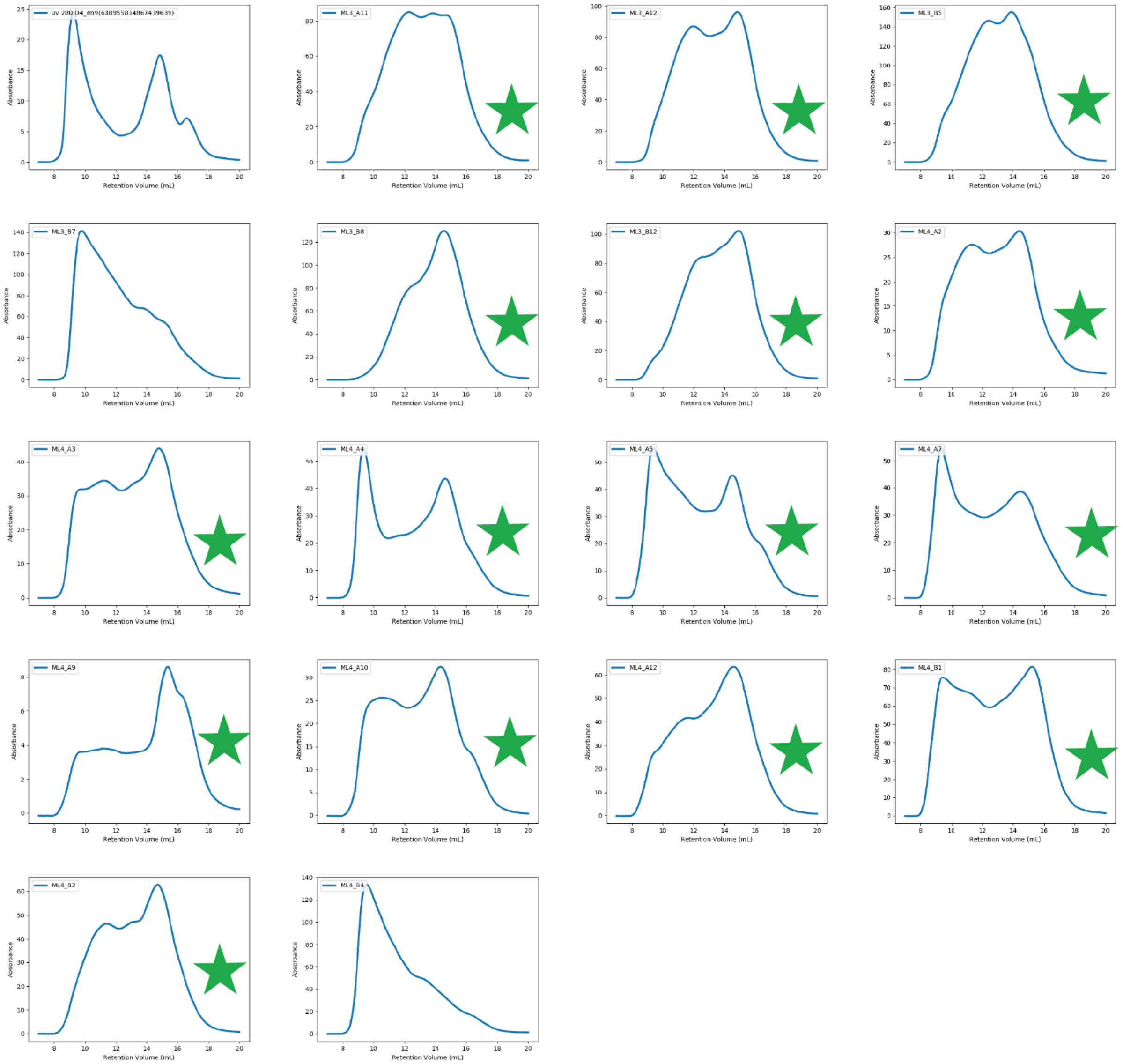

uv 280 D4_eb9(638955834867439639)
ML3_A11
ML3_A12
ML3_B5
ML3_B7
ML3_B8
ML3_B12
ML4_A2
ML4_A3
ML4_A4
ML4_A5
ML4_A7
ML4_A9
ML4_A10
ML4_A12
ML4_B1
ML4_B2
ML4_B4
Absorbance
Retention Volume (mL)

n16s

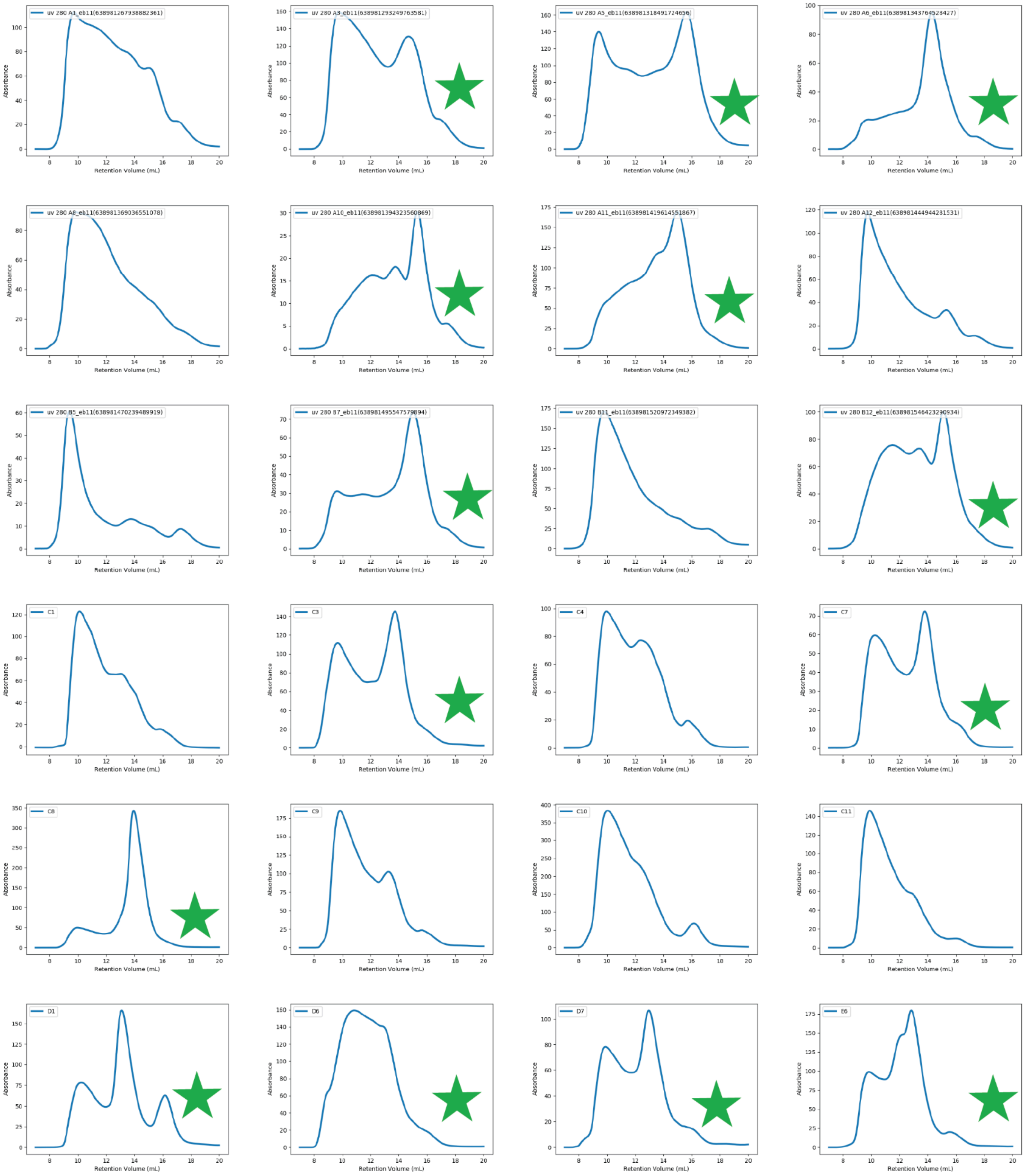

uv 280 A1_eb11(638981267938882361)
uv 280 A3_eb11(638981293249763581)
uv 280 A5_eb11(638981318491724656)
uv 280 A6_eb11(638981343764528427)
uv 280 A8_eb11(638981369036551078)
uv 280 A10_eb11(638981394323560869)
uv 280 A11_eb11(638981419614551867)
uv 280 A12_eb11(638981444944281531)
uv 280 B5_eb11(638981470239489919)
uv 280 B7_eb11(638981495547579894)
uv 280 B11_eb11(638981520972349382)
uv 280 B12_eb11(638981546423290934)
C1
C3
C4
C7
C8
C9
C10
C11
D1
D6
D7
E6
Absorbance
Retention Volume (mL)

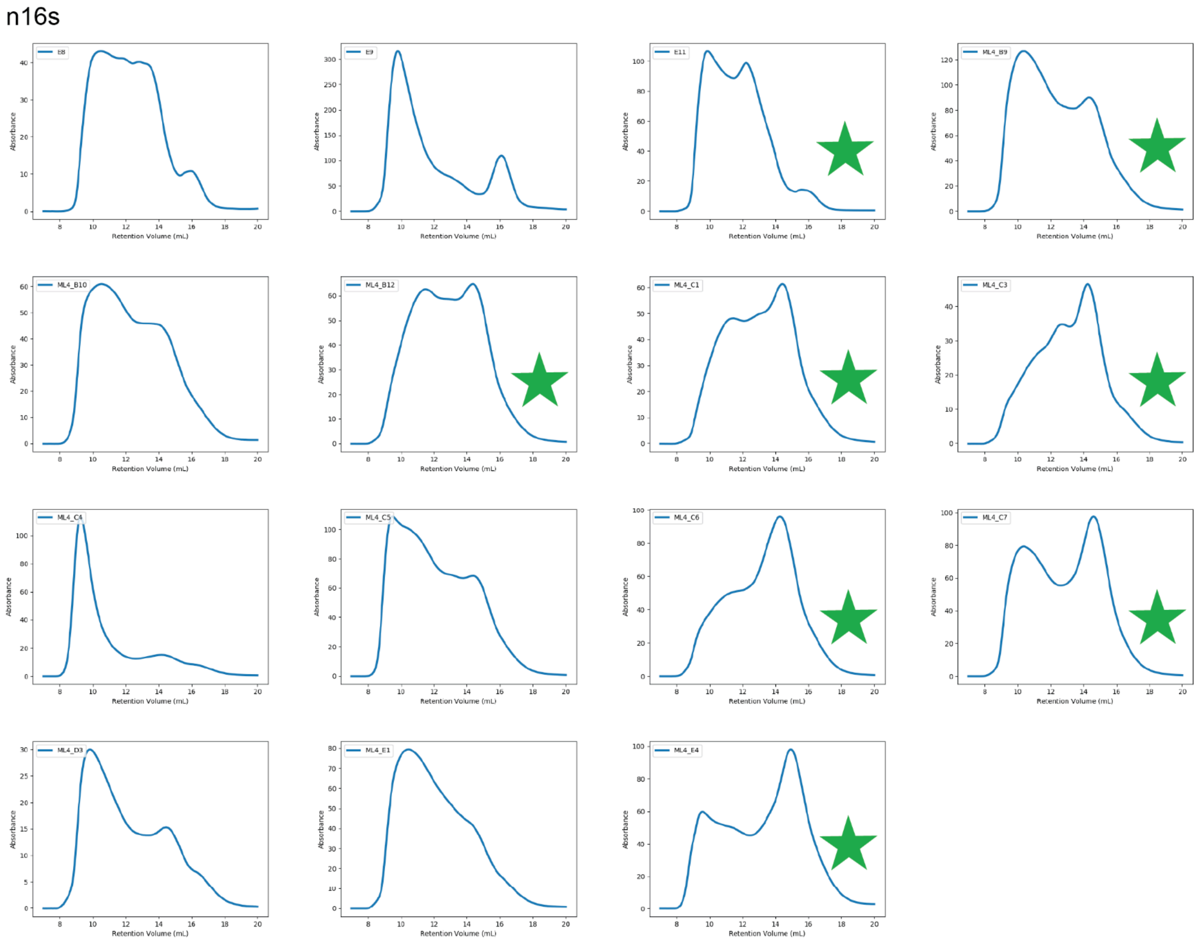

Figure S17. SEC traces of TMB_RFD2 and TMB_MPNN designs. Constructs with “good” traces (marked by green stars) were advanced to membrane insertion assays and used to generate Table 1.

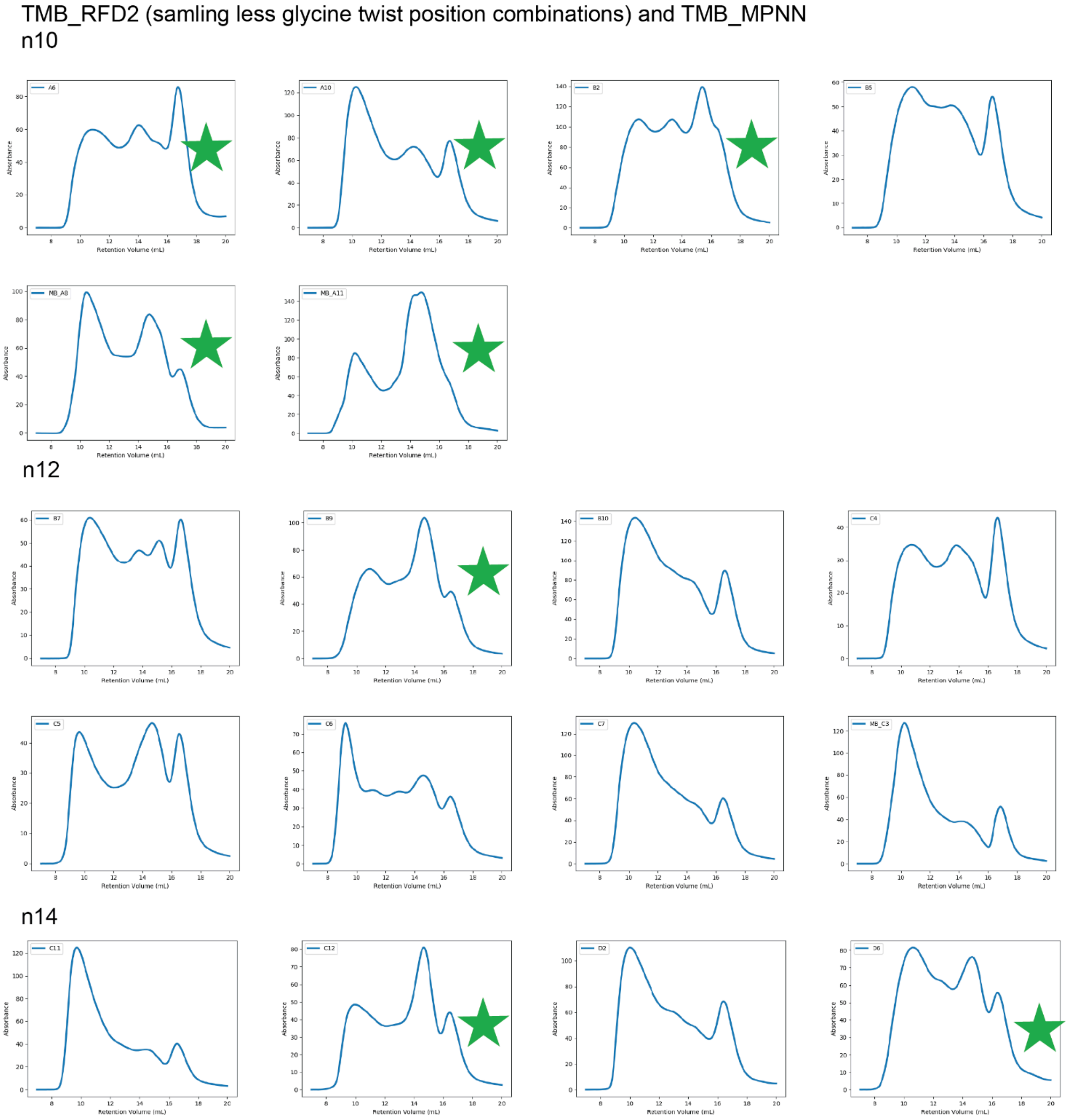

Figure S18. SEC traces of TMB_RFD2 and TMB_MPNN designs. Constructs with "good" traces (marked by green stars) were advanced to membrane insertion assays and used to compile Supplementary Table 3.

long_n10s

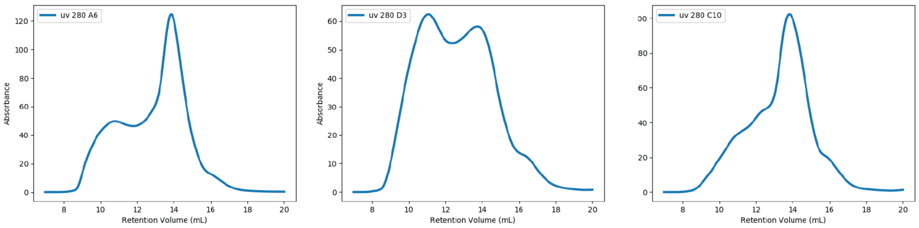

histidine_motif_design

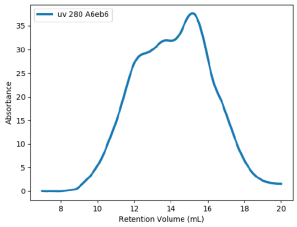

Figure S19. SEC traces of longer designs in Figure 6 and histidine motif design in figure 4.

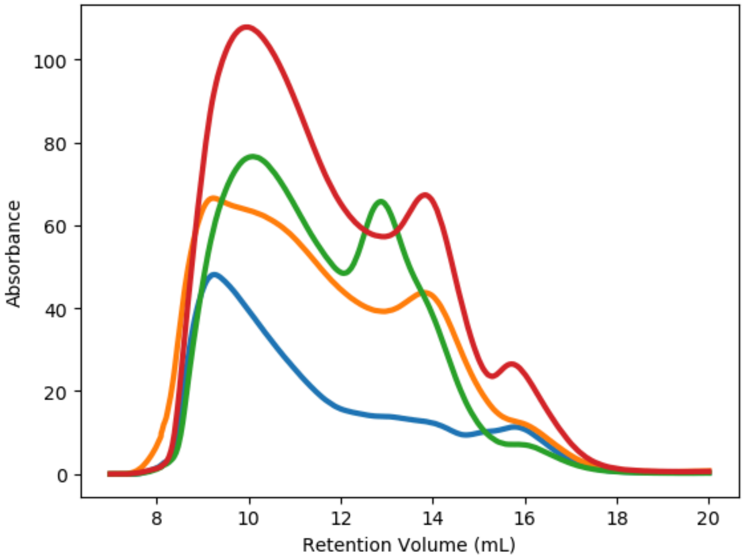

Figure S20. SEC of 18 stranded TMB designs

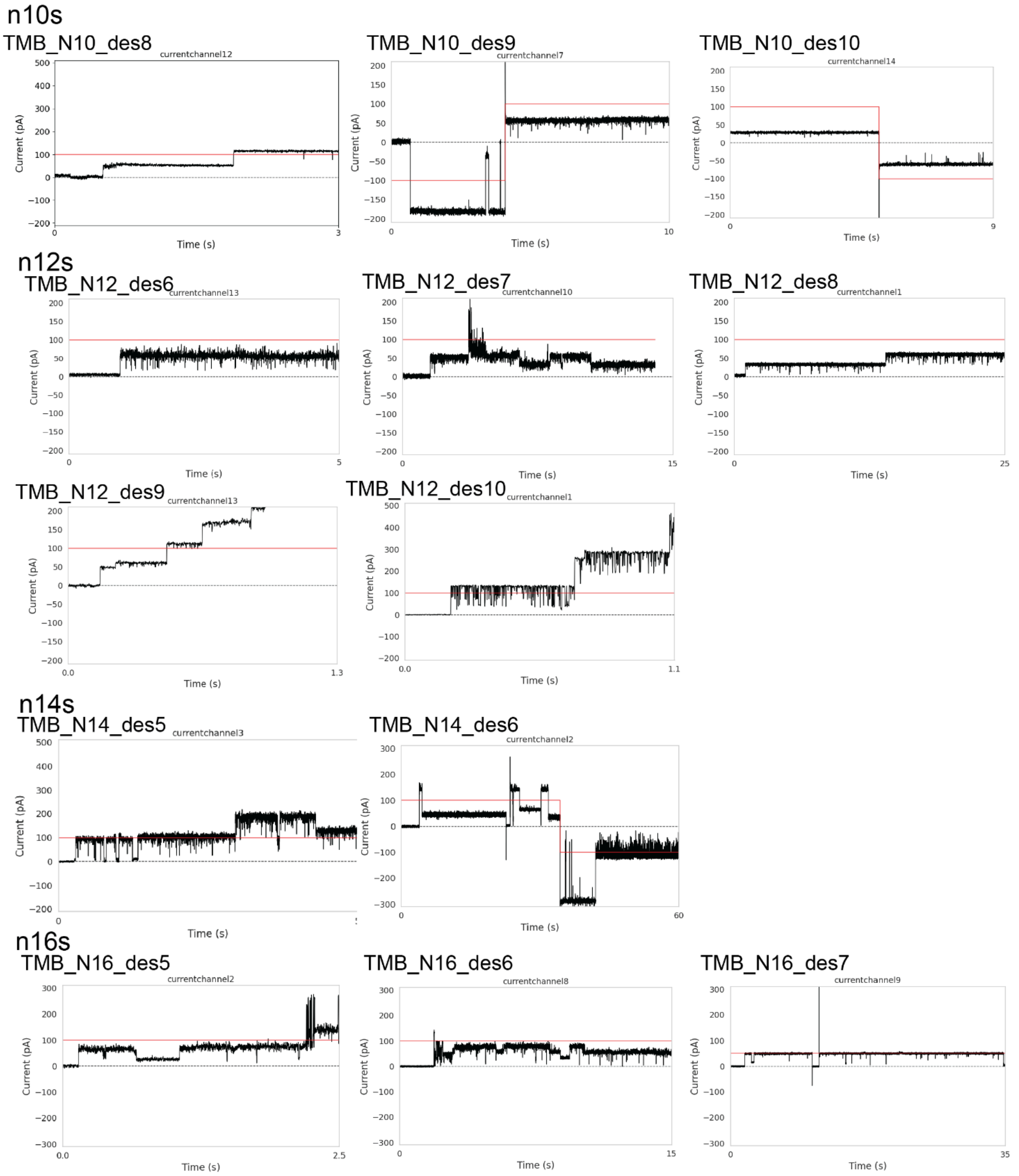

Figure S21. Additional functional designs from NC_TMB_RFD2 not shown in Figure 3. Observed conductance in 1M KCl.

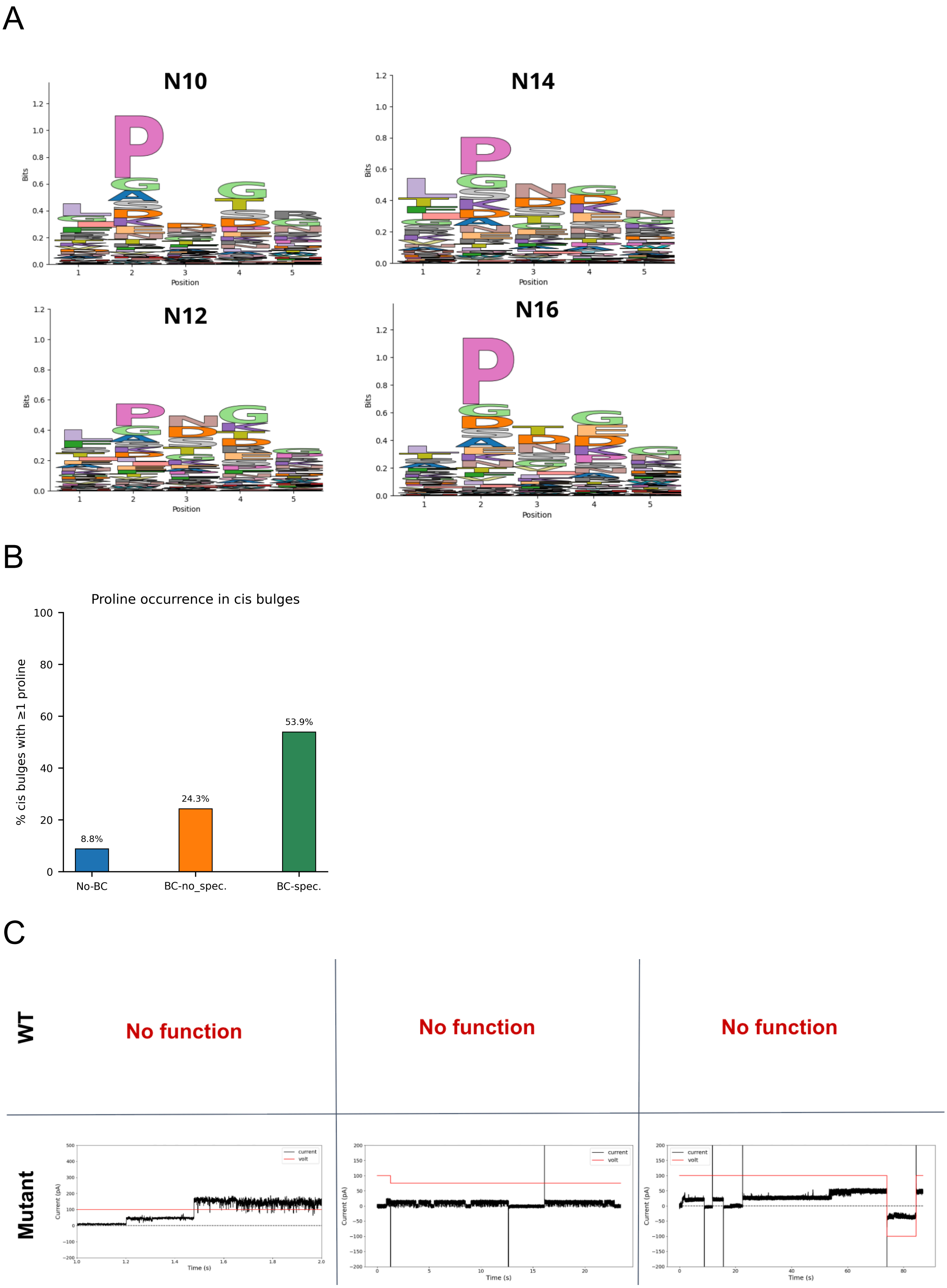
A
N10
N14
N12
N16
Bits
Position
B
Proline occurrence in cis bulges
% cis bulges with ≥1 proline
8.8%
24.3%
53.9%
No-BC
BC-no_spec.
BC-spec.
C
WT
Mutant
No function
No function
No function
current
volt
Current (pA)
Time (s)

Figure S22. (A) Sequence logo plots showing that proline is enriched at the second position of cis bulges across strand numbers 10, 12, 14, and 16. **(B)** Percentage of cis bulges with at least one proline and average bulges per structure both increase after conditioning. **(C)** Insertion tests showing that point mutations introducing prolines at cis bulge positions in previously nonfunctional designs converted them into functional pores capable of conducting ionic current.

n10s

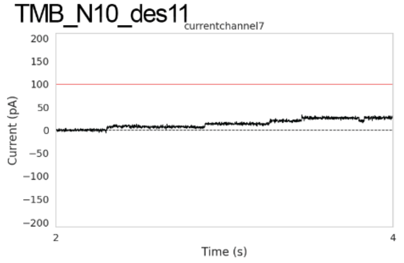

n12s

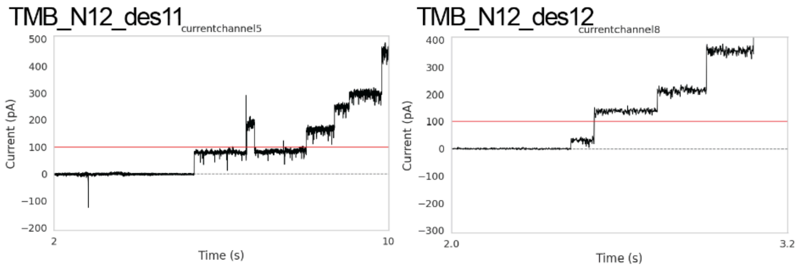

n14s

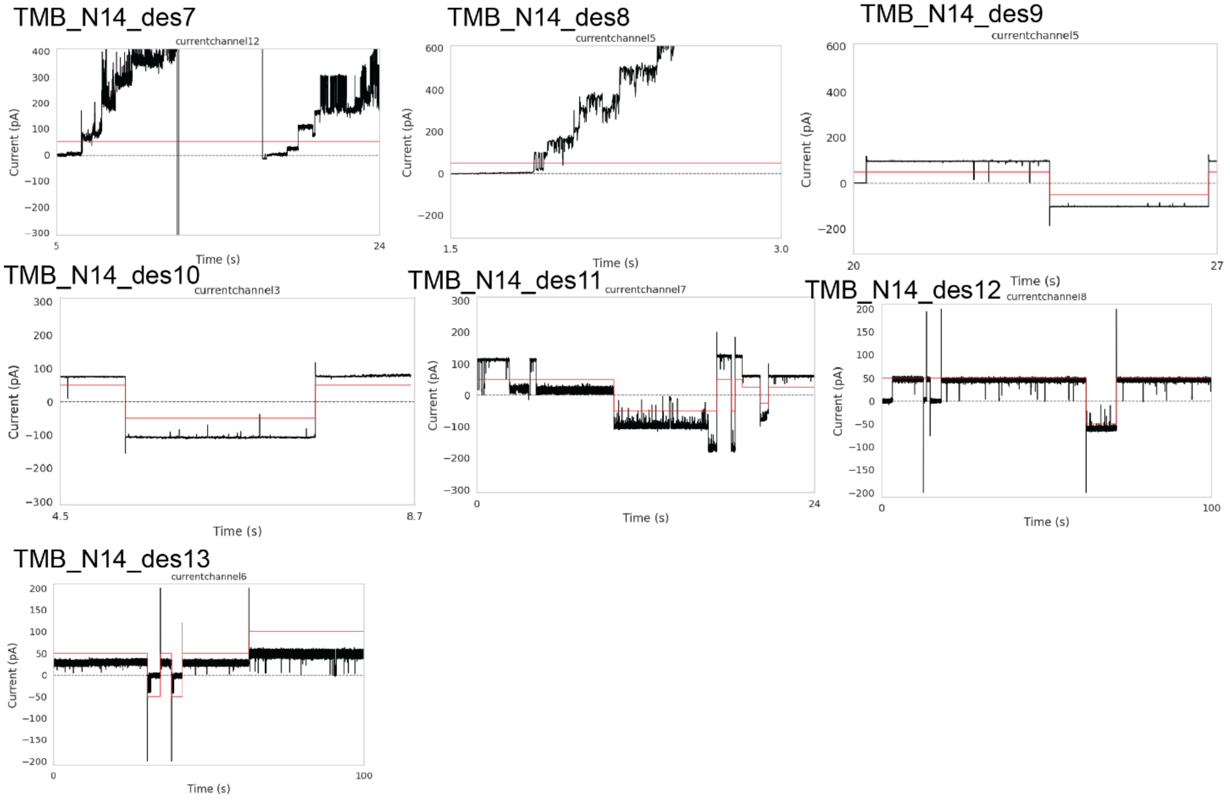

n16s

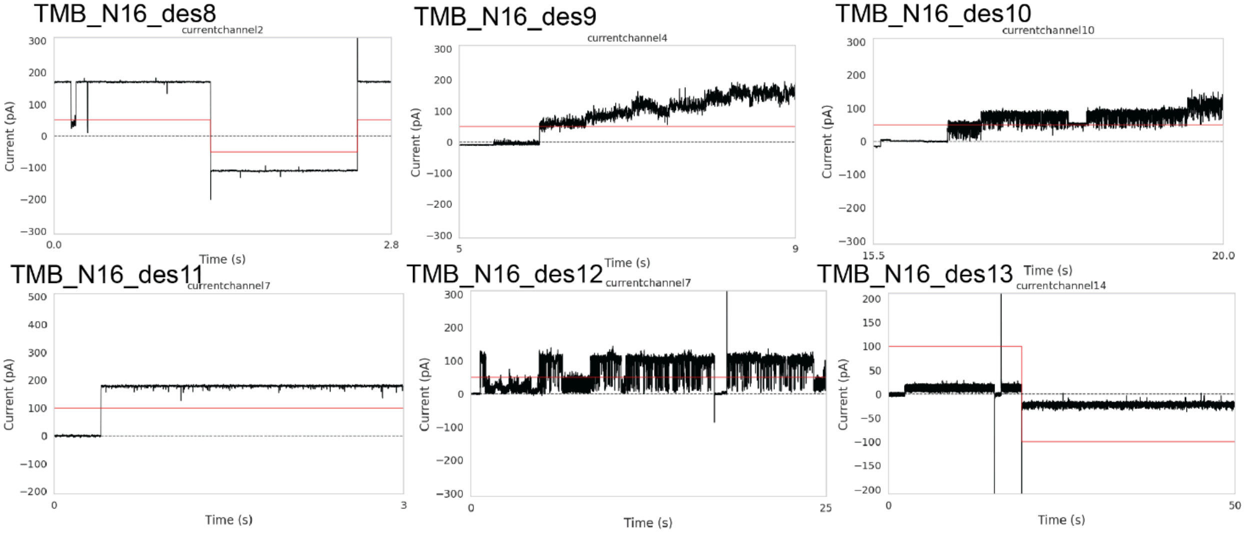

Figure S23. Additional functional designs from TMB_RFD2 not shown in Figure 3. Observed conductance in 1.5M KCl for 10 and 12 stranded designs and in 1M KCl for 14 and 16 stranded designs.

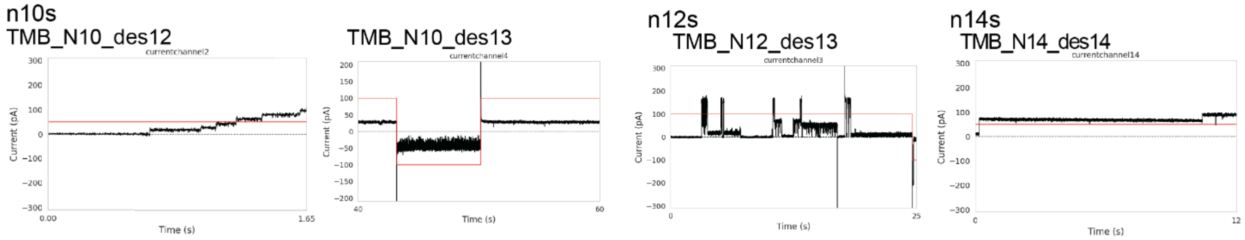

Figure S24. Functional designs from TMB_RFD2 with less glycine twist position combinations sampled at inference. Observed conductance in 1M KCl.

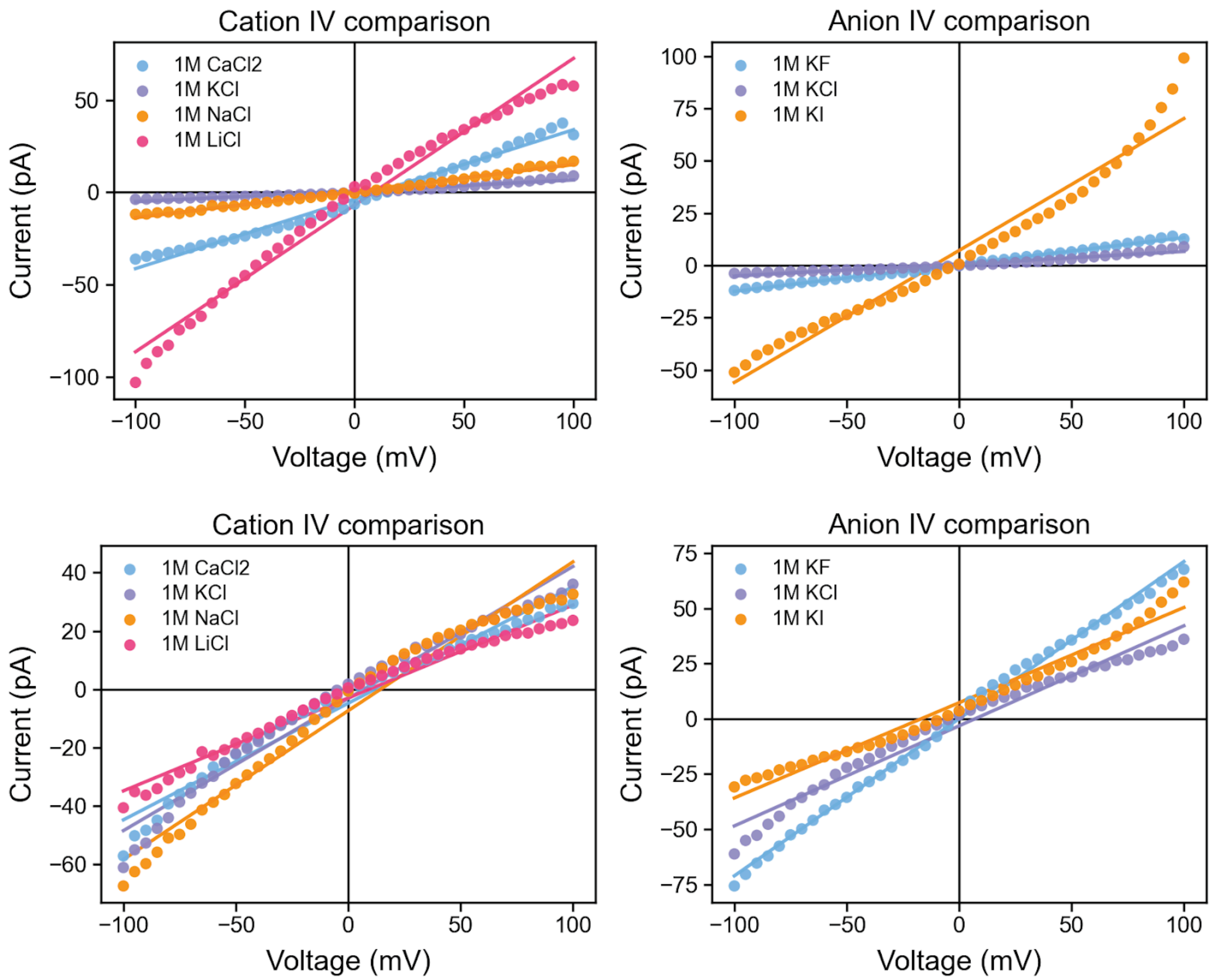

Figure S25: Conductance plots of an n10 and n12 with different cations and anions used in Figure 3b.

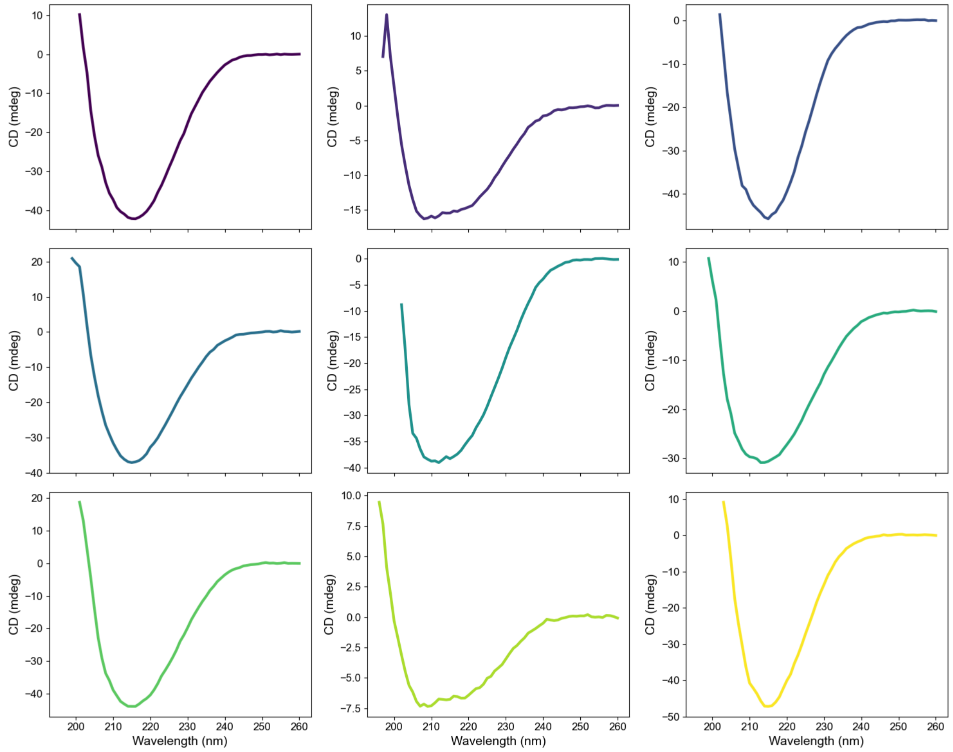

Figure S26. Circular dichroism (CD) for designs in order from left to right each row: N12_AP_des1, N16_RS_des1, N10_AP_des7, N14_AP_des2, N12_RS_des2, N14_AP_des1, N12_AP_des4, N16_RS_des5, N10_RS_des4

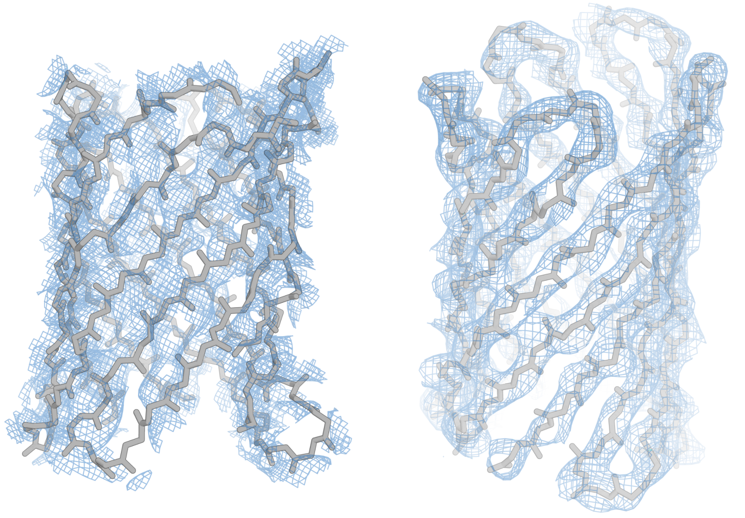

Figure S27. N10_RS_des4 (left) and N10_AP_des7 (right) are shown, with their main chains as grey sticks. 2mFo-DFc electron density maps contoured at 1.5 σ are shown in blue.

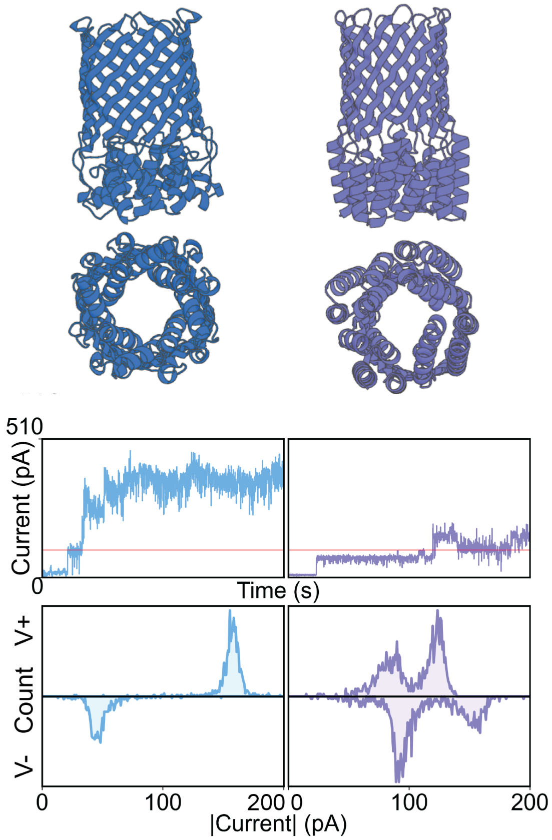

Figure S28. Symmetry-enforced multi-domain fusions. Conductance in 1MKCl and histograms for single channel recordings.

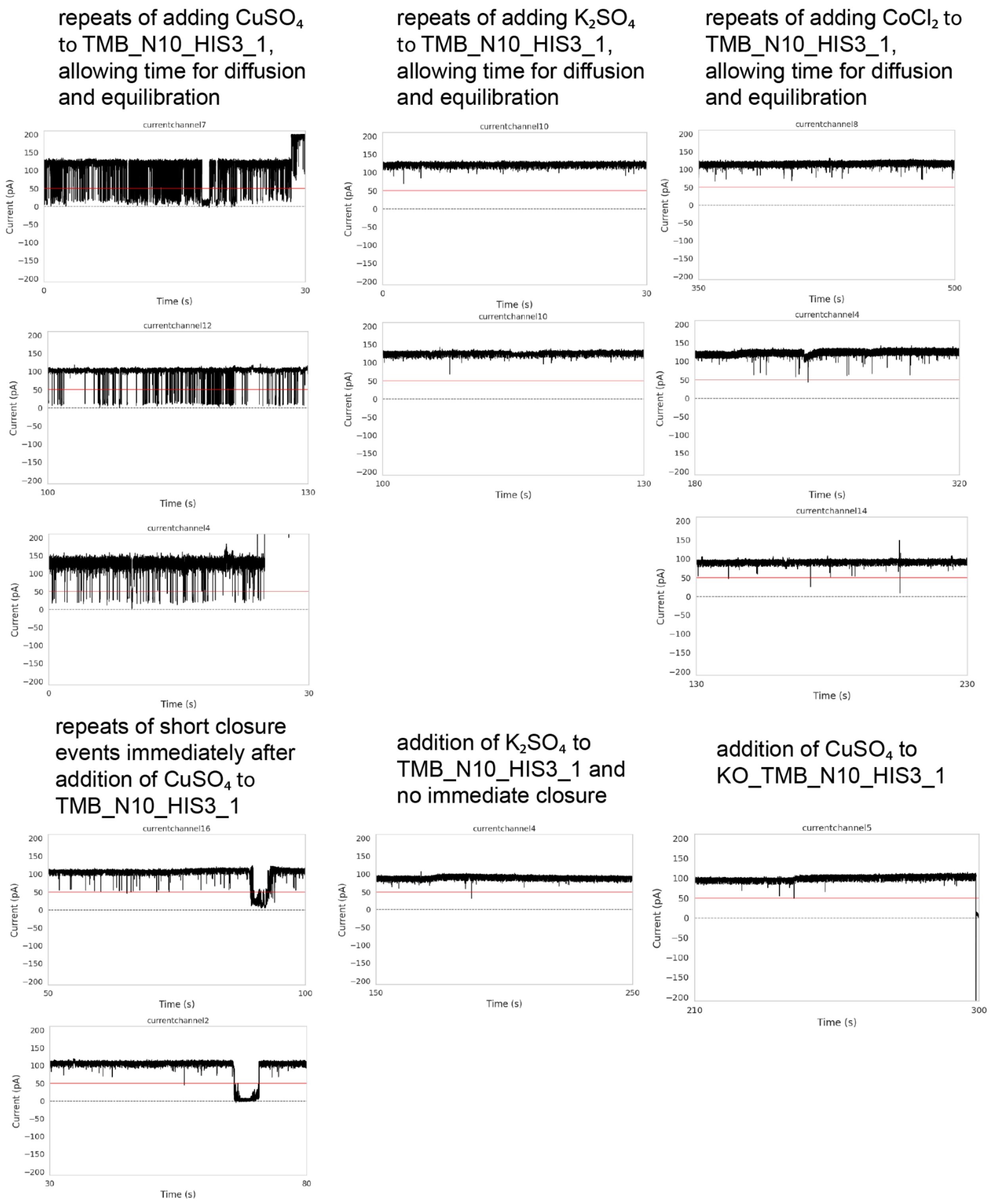

Figure S29. Repeats of behavior of histidine motif design, TMB_N10_HIS3_1, in different conditions. Current trace of KO_TMB_N10_HIS3_1 after addition of $CuSO_4$.

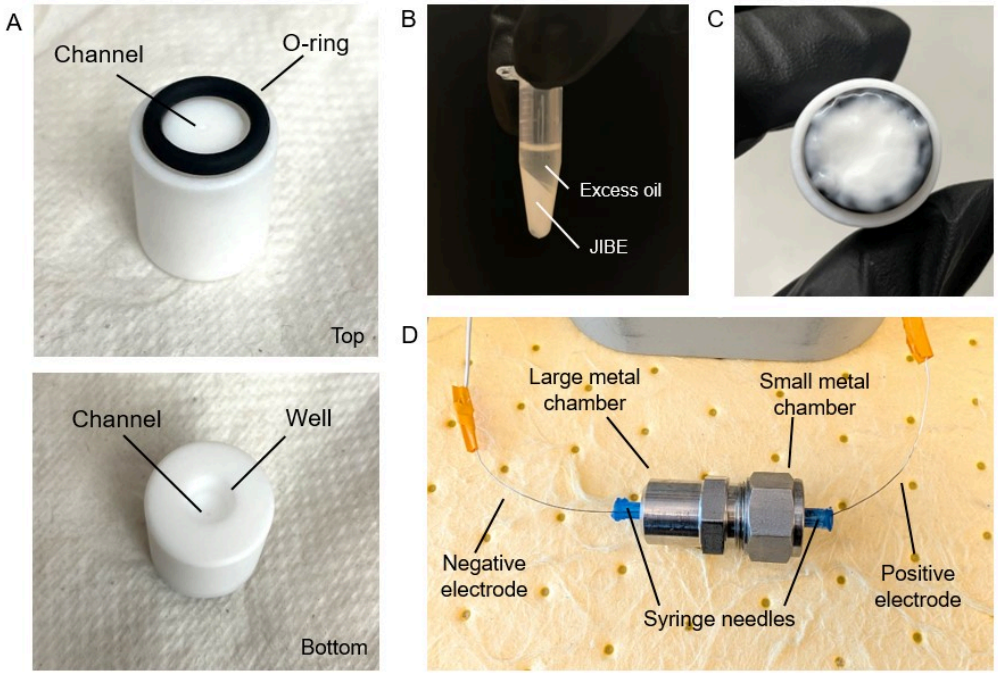

Figure S30. Overview of JIBE setup with agarose paddings. (A) Top and bottom views of plastic chamber with O-ring. (B) JIBEs form with an excess oil layer, which is pipetted off before adding to chamber setup. (C) JIBEs covering the surface of agarose pad and O-ring. (D) Fully assembled electrode and chamber setup.

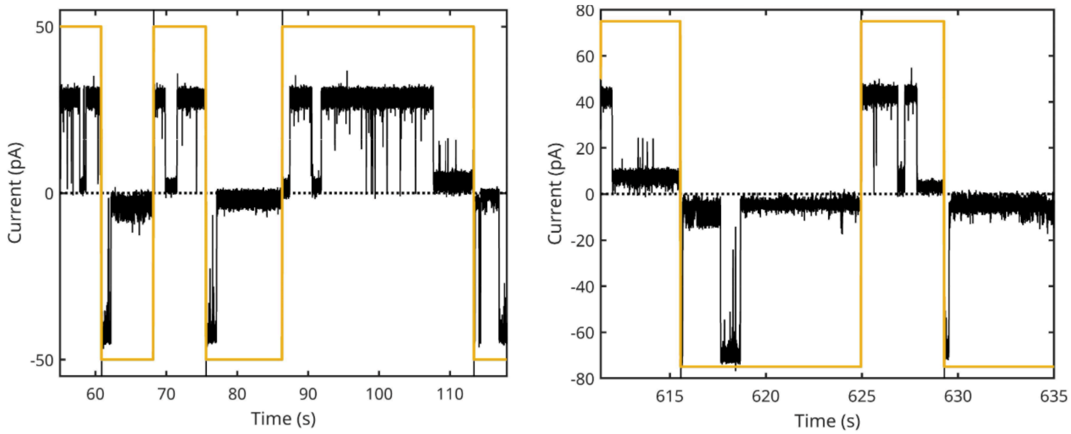

Figure S31. Stochastic gating observed in a design at 50mV and 75mV applied (dark yellow lines)

References:

1. Varadi, M. *et al.* AlphaFold Protein Structure Database in 2024: providing structure coverage for over 214 million protein sequences. *Nucleic Acids Res.* **52**, D368–D375 (2024).
2. Watson, J. L. *et al.* De novo design of protein structure and function with RFdiffusion. *Nature* **620**, 1089–1100 (2023).
3. Kabsch, W. XDS. *Acta Crystallogr. D Biol. Crystallogr.* **66**, 125–132 (2010).
4. Winn, M. D. *et al.* Overview of the CCP4 suite and current developments. *Acta Crystallogr. D Biol. Crystallogr.* **67**, 235–242 (2011).
5. McCoy, A. J. *et al.* Phaser crystallographic software. *J. Appl. Crystallogr.* **40**, 658–674 (2007).
6. Wojdyr, M., Keegan, R., Winter, G. & Ashton, A. *DIMPLE* - a pipeline for the rapid generation of difference maps from protein crystals with putatively bound ligands. *Acta Crystallogr. A* **69**, s299–s299 (2013).
7. Adams, P. D. *et al.* PHENIX: a comprehensive Python-based system for macromolecular structure solution. *Acta Crystallogr. D Biol. Crystallogr.* **66**, 213–221 (2010).
8. Emsley, P. & Cowtan, K. Coot: model-building tools for molecular graphics. *Acta Crystallogr. D Biol. Crystallogr.* **60**, 2126–2132 (2004).
9. Williams, C. J. *et al.* MolProbity: More and better reference data for improved all-atom structure validation. *Protein Sci. Publ. Protein Soc.* **27**, 293–315 (2018).
10. Murshudov, G. N. *et al.* REFMAC5 for the refinement of macromolecular crystal structures. *Acta Crystallogr. D Biol. Crystallogr.* **67**, 355–367 (2011).
11. Smart, O. S. *et al.* Exploiting structure similarity in refinement: automated NCS and target-structure restraints in BUSTER. *Acta Crystallogr. D Biol. Crystallogr.* **68**, 368–380 (2012).

12. Joosten, R. P., Long, F., Murshudov, G. N. & Perrakis, A. The PDB_REDO server for macromolecular structure model optimization. *IUCrJ* **1**, 213–220 (2014).
